# Cytokinin downregulates Photosystem II photochemistry during prolonged darkness in a phytochrome B‐dependent manner

**DOI:** 10.1111/nph.71224

**Published:** 2026-04-24

**Authors:** Veronika Kábrtová, Zuzana Kučerová, Ivo Chamrád, René Lenobel, Pavel Roudnický, Jan Skalák, Martin Hudeček, Filip Zavadil Kokáš, Marek Rác, Tereza Vánská, Jaroslav Nisler, Jan Hejátko, Miroslav Strnad, Martina Špundová, Ondřej Plíhal

**Affiliations:** ^1^ Laboratory of Growth Regulators, Institute of Experimental Botany Czech Academy of Sciences Šlechtitelů 27 779 00 Olomouc Czech Republic; ^2^ Laboratory of Growth Regulators, Faculty of Science Palacký University Šlechtitelů 27 779 00 Olomouc Czech Republic; ^3^ Department of Biophysics, Faculty of Science Palacký University Šlechtitelů 27 779 00 Olomouc Czech Republic; ^4^ CEITEC – Central European Institute of Technology, Mendel Centre of Plant Genomics and Proteomics Masaryk University Kamenice 5 625 00 Brno Czech Republic; ^5^ CEITEC – Central European Institute of Technology and National Centre for Biomolecular Research Masaryk University Kamenice 5 625 00 Brno Czech Republic; ^6^ Research Centre for Applied Molecular Oncology Masaryk Memorial Cancer Institute Žlutý kopec 7 656 53 Brno Czech Republic; ^7^ Isotope Laboratory Institute of Experimental Botany of the Czech Academy of Sciences Vídeňská 1083 142 20 Prague Czech Republic

**Keywords:** Chl, cytokinin, light, photosynthesis, Photosystem II, phytochrome, senescence

## Abstract

- Cytokinins (CKs) delay dark‐induced senescence, but how they tune photosynthetic function in darkness remains unclear.
- We investigated the effects of classical aromatic CK benzylaminopurine and CK‐derived compound 1‐(2‐methoxyethyl)‐3‐(1,2,3‐thiadiazol‐5‐yl)urea on detached Arabidopsis leaves at different time points during dark incubation. Notably, while both compounds mitigated the progression of senescence, they unexpectedly downregulated Photosystem II (PSII) photochemistry during the early stages of dark incubation. Despite this downregulation, CK‐treated leaves preserved functional photochemistry and substantially delayed Chl degradation at later time points.
- Transcriptomic analysis at the early stage of darkening revealed that a significant portion of CK‐responsive genes is associated with photosynthesis, PSII function, and light sensing, including red‐light signaling pathways. Differential proteomics further supported a CK‐induced shift to a metabolically suppressed state.
- The early PSII downregulation required phytochrome B, indicating a CK–phyB module that places darkened leaves into a reversible ‘standby’ mode, limiting energy demand and enabling rapid, safer recovery of photosynthesis upon re‐illumination. At the same time, it may also protect the photosynthetic apparatus against photo‐oxidative damage during the transition from dark‐ to light‐adapted states.

## Graphical abstract

Cytokinin–phyB signaling modulates PSII photochemistry during prolonged darkness.

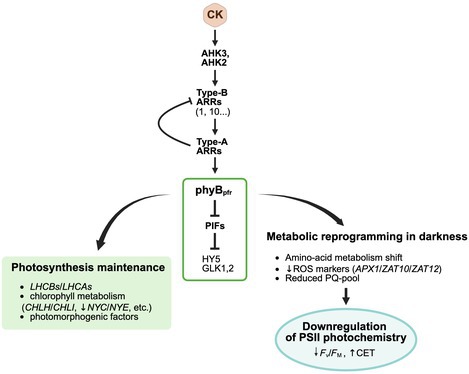

## Introduction

Cytokinins (CKs), key plant hormones, are pivotal in regulating cell division, differentiation, and numerous developmental and stress‐related responses (Schaller *et al*., 2014; Cortleven & Schmülling, 2015; Kieber & Schaller, 2018). A particularly well‐documented and notable function of CKs lies in their ability to delay the onset of senescence, which is associated with Chl retention in leaves with elevated CK levels (reviewed in Zwack & Rashotte, 2013; Hönig *et al*., 2018).

Beyond Arabidopsis, the regulation of photosynthetic efficiency by CKs has been studied in several crop species. CK‐mediated delay of senescence extends photosynthetic lifespan and maintains nutrient uptake, and elevated CK levels also affect fruit and seed development (Rivero *et al*., 2007; Jameson & Song, 2016). Consequently, either exogenous CK applications or genetic manipulations of CK biosynthetic/turnover represent a promising tool to improve agronomic traits (Plíhal *et al*., 2013; Pospíšilová *et al*., 2016). Understanding the mechanisms by which CKs sustain photosynthetic activity and coordinate stress protection, therefore, has broad implications for improving crop resilience under fluctuating light and environmental stress conditions (Hudeček *et al*., 2022).

CKs are predominantly synthesized in root vasculature via the isopentenyl transferase (IPT) pathway, which produces isopentenyladenine (iP)‐type CKs, and through tRNA degradation, which generates *cis*‐zeatin (*c*Z)‐type forms in the root apices (Kasahara *et al*., 2004; Antoniadi *et al*., 2015). The biologically active free bases are released from inactive nucleotides by LONELY GUY (LOG) phosphoribohydrolases, whereas their irreversible degradation is catalyzed by cytokinin oxidase/dehydrogenase (CKX) enzymes (Zürcher & Müller, 2016).

Perception of CKs occurs through a conserved multistep phosphorelay system structurally analogous to the bacterial two‐component signaling pathway. In Arabidopsis, three hybrid histidine kinase receptors – AHK2, AHK3, and CRE1/AHK4 – mediate CK perception via the cyclase/histidine kinase‐associated sensory extracellular (CHASE) domain (Inoue *et al*., 2001; Suzuki *et al*., 2001; Ueguchi *et al*., 2001). Ligand perception triggers autophosphorylation, followed by a His‐Asp phosphorelay involving phosphotransfer proteins (AHPs) and type‐B response regulators (ARRs), which act as transcriptional activators of CK‐responsive genes (Brenner *et al*., 2012; Heyl *et al*., 2012). The signaling cascade is fine‐tuned by rapidly induced, DNA‐binding–deficient type‐A ARRs (e.g. *ARR4*, *ARR5*, *ARR7*, and *ARR16*), which provide negative feedback regulation to attenuate the CK‐driven transcriptional response (To *et al*., 2004). Expression of type‐A *ARRs* has therefore been widely used as a proxy for CK pathway activation (D'Agostino *et al*., 2000). However, type‐A *ARR* genes are not exclusively regulated by CK, but are also responsive to other environmental and hormonal cues. Intriguingly, recent findings by Gautrat *et al*. (2024) revealed that PIF7, together with other PHYTOCHROME‐INTERACTING FACTORS (PIFs), directly represses the transcription of fast‐response type‐A *ARRs* (*ARR4*, *ARR5*, and *ARR6*; Gautrat *et al*., 2024). Their study further showed that root‐derived *t*Z enhances hypocotyl elongation only under far‐red (FR)‐enriched light conditions, where phyB is inactive, which in turn leads to attenuation of type‐A *ARR* feedback by PIFs.

Although the three AHK receptors display partially redundant functions, they exhibit distinct ligand preferences, expression profiles, and physiological roles (Spíchal *et al*., 2004; Riefler *et al*., 2006). AHK3 seems to be predominantly active in shoot tissues, where it plays a key role in maintaining Chl content and delaying senescence (Kim *et al*., 2006). While the basal synthesis of Chl can occur independently of CK signaling, AHK2 and particularly AHK3 are required to sustain wild‐type (WT) levels of Chl, as demonstrated by the *ahk2 ahk3* loss‐of‐function mutants (Riefler *et al*., 2006). Interactions between CK signaling and light‐regulated transcription factors, such as GLK1/GLK2, HY5, GNC, and CGA1 have been proposed, suggesting the existence of a shared regulatory platform integrating CK, light, and other hormonal pathways (Cortleven & Schmülling, 2015).

At the level of the photosynthetic apparatus, CKs partially mimic the effects of light and regulate photomorphogenesis, particularly in dark conditions (Chory *et al*., 1994; Lochmanová *et al*., 2008). Beyond affecting chloroplast ultrastructure, CKs (including aromatic CK 6‐benzylaminopurine (BAP)) help maintain photosynthetic function, including Photosystem II (PSII) photochemistry in stressed and senescing leaves (Vlčková *et al*., 2006; Vylíčilová *et al*., 2016; Janečková *et al*., 2019; Nisler *et al*., 2023).

The antisenescence properties of many CKs and CK‐derived compounds have been previously studied (Mik *et al*., 2011; Vylíčilová *et al*., 2016; Nisler *et al*., 2018; Kučerová *et al*., 2020). The antisenescence effects of CKs typically become apparent after 4–6 d after detachment and darkening (DAD), when Chl levels in mock‐treated leaves decrease dramatically (Vlčková *et al*., 2006; Janečková *et al*., 2019; Kučerová *et al*., 2020). Here, we describe a previously unreported effect of exogenous CK in detached leaves incubated in the dark for a shorter period (2 DAD): PSII photochemistry was significantly downregulated in CK‐treated leaves compared with mock controls. Further experiments showed that this CK‐mediated downregulation of PSII photochemistry was dependent on phytochrome B (phyB).

The principal red/FR photoreceptor, phyB, coordinates photomorphogenesis and metabolic transitions between light and dark states through interaction with PIFs and ELONGATED HYPOCOTYL 5 (HY5; Wang & Wang, 2015). Recent evidence indicates that phyB also influences chloroplast development, carbon metabolism, and hormonal cross‐talk during stress adaptation (Yoo *et al*., 2019). However, it is still unknown whether CK signaling converges with phyB‐dependent pathways to modulate photosynthetic functions during changes in light conditions.

Our results provide new insights into the poorly understood CK‐phytochrome signaling crosstalk during the initial phase of darkening, before the onset of dark‐induced leaf senescence. For this study, we used the canonical CK BAP and a new synthetic compound with low CK activity but pronounced antisenescence effects, MTU (1‐(2‐methoxyethyl)‐3‐(1,2,3‐thiadiazol‐5‐yl)urea; Nisler *et al*., 2018; Nisler *et al*., 2023). MTU (previously referred to as ASES in Nisler *et al*., 2018) has demonstrated *c*. 100‐fold higher activity than BAP in a wheat leaf senescence assay and appears to act through both CK‐dependent mechanisms – via weak interaction with AHK3 – and additional, CK‐independent pathway(s) (Nisler *et al*., 2018).

## Materials and Methods

### Plant material, growth conditions, and experimental procedures

Experiments were performed using *Arabidopsis thaliana* (L.) Heynh ecotype Columbia‐0 (Col‐0) (WT) and its mutants listed below. Seeds of knockout mutant lines *ahk2‐2 ahk3‐3*, *ahk2‐5 cre1‐2*, and *ahk3‐7 cre1‐2* (Riefler *et al*., 2006) were kindly provided by Prof. Thomas Schmülling (Free University of Berlin, Germany). Seeds of knockout mutants *phyA* (SALK_014575C) and *phyB* (SALK_022035C) were obtained from Nottingham Arabidopsis Stock Centre (Alonso *et al*., 2003), and T‐DNA insertion was verified by PCR genotyping. Seeds of *arr1*, *arr2*, *arr10*, *arr12*, and *arr1arr12* double mutants were provided by Prof. M. Umeda (Nara Institute of Science and Technology, Japan), and *arr4* mutant was provided by Dr M. Pernisová (MUNI, Brno, Czech Republic). Seeds of *35S::PHYB:GFP* line (Rausenberger *et al*., 2010) were provided by Prof. A. Hiltbrunner (University of Freiburg, Germany).

Plants were grown in a soil (Potgrond H, Klasmann‐Deilmann, Geeste, Germany) in a growth chamber under an 8 h : 16 h, 22°C : 20°C, light : dark cycle (110 μmol photons m^−2^ s^−1^; LED). For all experiments (except for qPCR analysis), 33‐d‐old plants were used. In the case of qPCR analysis, 6‐wk‐old plants were used because a larger quantity of plant material was required for mRNA isolation. The seventh–ninth rosette leaves were detached from the plants and incubated in 0.2% dimethylsulfoxide (DMSO) in deionized water as a mock control or in a solution of BAP or MTU (for synthesis see Nisler *et al*. (2018)) at concentrations of 0.5 or 5 μM in 0.2% DMSO. Leaves in described solutions were kept in the dark at 21°C for 6 h or 2, 5, 6, 7, 9, or 12 d after detachment and darkening (DAD) depending on the experiment. Freshly detached leaves were used as a control (0 DAD). In the case of FR treatment, the leaves were detached, darkened, and subsequently illuminated by FR light (20 μmol photons m^−2^ s^−1^) using CG‐RG‐715‐50.0 M‐2.5 filter (CVI Melles Griot, Carlsbad, CA, USA) in combination with white LED light for 15 min while floating in deionized water. After the FR illumination, the leaves were transferred into solutions described above and kept in the dark at 21°C for 2 d.

In the case of experiments with intact plants, the Arabidopsis WT and *phyB* (SALK_022035C) plants were grown hydroponically according to Conn *et al*. (2013), for 33 d in a growth chamber under an 8 h : 16 h, 22°C : 20°C, light : dark cycle (110 μmol photons m^−2^ s^−1^; LED). Afterward, the growing solution was replaced with 0.02% DMSO in deionized water (mock control) or in a solution of 5 or 10 μM BAP/MTU (in 0.02% DMSO). Plants were kept in the described solutions in the dark for 1, 3, or 10 d.

### Determination of Chl*a* + *b* content and Chl*a* : Chl*b* ratio

To determine the leaf area, detached leaves were traced onto a transparent film, which was then scanned, and the resulting area was calculated using the ImageJ software (National Institute of Health, Bethesda, MD, USA). Next, the leaves were frozen in liquid nitrogen, stored at −80°C, and subsequently homogenized using 80% acetone and a small amount of MgCO_3_. The homogenate was centrifuged at 6000 ***g*** for 10 min at 4°C. The supernatant was used for the determination of Chl *a* and Chl *b* content according to Lichtenthaler (1987). The sum of Chl *a* + *b* and the ratio of Chl*a* : Chl*b* were estimated and presented.

### Photosynthetic parameters (PSII and PSI functioning)

Chl fluorescence quenching analysis was measured from the adaxial side of the leaves using the FluorCam FC800‐O imaging system (PSI, Drásov, Czech Republic) and the DualPam100 measuring system (Heinz Walz, Effeltrich, Germany). The freshly detached leaves used as control were dark‐adapted before measurement for 25 min.

FluorCam imaging system was used for measurement of the maximum quantum yield of PSII photochemistry (*F*_V_/*F*_M_ = (*F*_M_–*F*_0_)/*F*_M_) and for measurement of quantum yields Φ_P_, Φ_NPQ_, and Φ_f,D_ as described previously (Kučerová *et al*., 2020), with slight modifications. The maximal fluorescence of the dark‐adapted sample (*F*_M_) was determined by applying an 800‐ms‐long saturating pulse (blue light, 3500 μmol photons m^−2^ s^−1^). For estimation of maximal fluorescence at light‐adapted state (*F*_M_′), the sample was exposed to actinic light (red light, 230 μmol photons m^−2^ s^−1^) and a series of saturating pulses. The last measured (steady‐state, i.e. after 10 min of the actinic light) values for quantum yields Φ_P_, Φ_NPQ_, and Φ_f,D_ are presented.

Parameters reflecting the electron transport rate through PSII (ETRII), PSI (ETRI), and cyclic electron transport (CET) were estimated using the DualPam100 measuring system during exposure to actinic light for 11 min (red light, 800 μmol photons m^−2^ s^−1^) and using 300‐ms‐long saturating pulses (red light, 10 000 μmol photons m^−2^ s^−1^) as described in Nisler *et al*. (2023).

### Proteomic analysis

Arabidopsis leaves incubated in 0.2% DMSO, BAP, or MTU solutions at 5 μM concentration were kept in the dark at 21°C for 6 h or 2 d. Leaves were documented and weighed, and quadruplicate samples comprising five leaves from five different plants were prepared and frozen in liquid nitrogen for each treatment variant. Leaf samples were then pulverized, and a hot sodium dodecyl sulfate (SDS)‐based buffer (5% SDS, 250 mM dithiotreitol, 250 mM Tris‐hydrochloric acid, pH 7.6) was added. Following 60 min of incubation at 95°C under continuous shaking, tissue debris was removed by centrifugation, and 10 μg of total protein was digested with trypsin following the FASP protocol (Wiśniewski *et al*., 2011). The resulting peptides were cleaned by phase transfer (Masuda *et al*., 2008), extracted into liquid chromatography mass spectrometry (LC‐MS) vials by 2.5% formic acid (FA) in 50% acetonitrile (ACN) and 100% ACN with the addition of polyethylene glycol (20 000; final concentration 0.001%; Stejskal *et al*., 2013), and concentrated in a SpeedVac concentrator (Thermo Fisher Scientific, Waltham, MA, USA).

The samples were subsequently analyzed by LC‐MS/MS using a nanoElute® system (Bruker, Billerica, MA, USA) connected online to a timsTOF Pro mass spectrometer (Bruker). The acquired raw Tims‐MS data were processed and analyzed using the Maxquant software v.1.6.17.0 (Tyanova *et al*., 2016), with built‐in Andromeda as a search engine (Cox *et al*., 2011). For protein identifications, a concatenated database combining *Arabidopsis thaliana* proteins (The UniProt Knowledgebase; taxon ID: 3702; 27 468 protein sequences; download: 2 December 2020) and the cRAP contaminant database (http://www.thegpm.org/crap/; v.181122; 112 protein sequences) was used. Protein searches were performed with a match between runs across the whole dataset to improve peptide‐spectra matching. Peptides and proteins with false discovery rate < 1% and at least one razor peptide were subjected to differential abundance analyses based on loessF normalized intensities using Knime (Berthold *et al*., 2006).

### Immunoblot analysis

Detached leaves were kept in the dark for 2, 5, or 7 d while incubated in 0.2% DMSO or 5 μM BAP/MTU and subsequently frozen in liquid nitrogen. Each sample consisted of five leaves from different plants. Leaves were homogenized in liquid nitrogen and mixed with protein extraction buffer (14 mM dithiothreitol, 28 mM Na_2_CO_3_, 175 mM sucrose, 5% SDS, and 10 mM Na_2_‐ethylenediaminetetraacetic acid (EDTA)), incubated at 70°C for 30 min, and centrifuged for 10 min at 19 200 ***g***. Isolated proteins (corresponding to 0.5 mg of sample FW) were diluted with sample buffer (62.5 mM Tris pH 6.8, 10% glycerol, 5% SDS, 5% β‐mercaptoethanol, and 0.01% bromophenol blue) and incubated at 70°C for 10 min. Subsequently, samples were loaded onto 10% gel (3 M Tris pH 8.45; acrylamide/bis‐acrylamide 29 : 1 ratio) and the electrophoresis was performed at RT with a constant voltage at 100 V (composition of anode buffer: 100 mM Tris pH 8.9; cathode buffer: 100 mM Tris, 100 mM tricine, 0.1% SDS, 0.5 M EDTA). The separated proteins were transferred onto a PVDF membrane by *Trans*‐Blot Turbo transfer system (Bio‐Rad). For immunoblot analyses, the specific antibodies anti‐Lhca1, anti‐Lhca2, anti‐Lhcb1, and anti‐Lhcb2 (Agrisera, Vännäs, Sweden) were used, and the chemiluminescent signal from the horseradish peroxidase (HRP)‐conjugated secondary antibody (Bio‐Rad) was detected using Immobilon chemiluminescent HRP substrate (Merck KGaA, Darmstadt, Germany) and gel scanner Amersham Imager 600RGB (GE HealthCare Life Sciences, Hino, Japan).

### Reactive oxygen species imaging by confocal microscopy

Detached leaves incubated in solutions of 0.2% DMSO or 5 μM BAP or MTU in the dark for 2 d were cut into segments (*c*. 2 × 2 mm) and underwent vacuum infiltration with 10 μM 2′,7′‐dichlorodihydrofluorescein diacetate (DCF‐DA; Merck KGaA) in 20% methanol inside of a syringe. Leaf segments were subsequently illuminated by high light (red light, 800 μmol photons m^−2^ s^−1^) for 15 min. The samples were visualized by confocal microscope BC43 (Oxford Instruments plc, Abingdon, UK) using the fluorescent probe DCF‐DA for imaging of reactive oxygen species (ROS). Fluorescence channel excitation was at 488 nm, and emission was detected at 517–541 nm. The proper intensity of the laser was set according to unstained samples at the beginning of each experiment.

### RNA‐seq analysis and data processing

To extract total RNA from the Arabidopsis leaves, we employed the Spectrum™ Plant Total RNA Kit (Merck, Burlington, MA, USA). The isolated RNA samples were then diluted to a 300 ng μl^−1^ concentration. To ensure the removal of any contaminating DNA, Turbo DNase treatment was conducted following the procedure outlined in the Turbo DNA‐free Kit (Thermo Fisher Scientific).

For the preparation of plant cDNA sequencing libraries, we used 4 μg of total RNA per sample and the Illumina TruSeq Stranded mRNA Sample Preparation Kit (Illumina, San Diego, CA, USA). The libraries were quantified by qPCR and pooled to achieve a final concentration of 14 pM. The NovaSeq 6000 S1 Reagent Kit was utilized for sequencing on the NovaSeq 6000 platform (Illumina). This comprehensive experimental workflow involved three independent libraries prepared from three biological replicates.

Low‐quality reads were removed from the raw sequencing data and adaptor sequences clipped, as well as leading or trailing regions of low quality (below 18 phred) using Trimmomatic v.0.33 package. Reads were mapped to the reference genome using TopHat2; the reference sequence version used was TAIR10 from ENSEMBL (Howe *et al*., 2021). Gene coverage was calculated using HTSeq‐count. Differential expression was evaluated using DESeq2.

### RT‐qPCR

For qPCR analysis, the leaves of 6‐wk‐old plants (Arabidopsis WT or *phyB* SALK_022035C) were used. Detached leaves were incubated in solutions of 0.2% DMSO or 5 μM BAP/MTU in the dark for 2 d, as in previous assays.

Total RNA was extracted as above and subjected to TurboDNase treatment. Subsequently, 2 μg of isolated and treated RNA was employed for cDNA synthesis, utilizing an oligo dT primer (Generi Biotech, Hradec Králové, Czech Republic) and Revert Aid Reverse Transcriptase (Thermo Fisher Scientific) according to the manufacturer's instructions.

The resulting cDNA was then diluted and combined with LightCycler 480 SYBR Green I Master (Roche). For reverse transcription quantitative polymerase chain reaction (RT‐qPCR), forward and reverse primers were used at a final concentration of 250 nM each (primer combinations outlined in Supporting Information Table S1). To assess the transcript abundance, the experiment was conducted using three independent biological replicates (each biological replicate consisted of at least 20 leaves from different plants), and each sample was run in four technical replicates within the CFX Opus 384 Real‐Time PCR System (Bio‐Rad). Genes coding for Elongation factor 1α (*EF1*α) and Actin 7 (*ACT7*) were used as reference genes for normalization using the 2^−*∆∆*Ct^ method (Livak & Schmittgen, 2001). Subsequently, the data collected from the experiment were analyzed in Excel, and statistical significance was determined using an unpaired Student's *t*‐test.

### Malondialdehyde (MDA) determination

Detached leaves that were kept in the dark for 2 d were subsequently illuminated by high light (white light, 800 μmol photons m^−2^ s^−1^) for 15 min, frozen in liquid nitrogen, and stored at −80°C. The leaves were homogenized in 1.5 ml of deionized water. Homogenate was centrifuged at 6000 ***g*** for 10 min at 4°C, and 100 μl of supernatant was taken. Total MDA was obtained by alkaline hydrolysis and protein precipitation (Pilz *et al*., 2000) with a few modifications. To achieve alkaline hydrolysis of protein‐bound MDA, 20 μl of 6 M aqueous sodium hydroxide was added to the sample and incubated in a dry bath at 60°C for 30 min (Thermo‐Shaker TS100; Biosan, Riga, Latvia). To reach the precipitation of proteins in samples, 60 μl of 35% (v/v) perchloric acid was added, vortexed, and centrifuged at 16 000 ***g*** for 10 min at 4°C. For the derivatization of MDA with DNPH, 100 μl of supernatant was taken into a vial, and 1 μl of 50 mM DNPH dissolved in 50% sulfuric acid was added and incubated in the dark at room temperature for 30 min. A volume of 10 μl of sample containing MDA‐DNPH adduct was injected into the high‐performance liquid chromatography (HPLC) system (Alliance e 2695 HPLC System; Waters, Milford, MA, USA), and the absorbance was detected at 310 nm using a PDA detector. An Astra C18‐HE (3 μm; 4.6 mm × 150 mm) column (Chromservis, Praha, Czech Republic) was used. The analysis was performed isocratically (1 ml min^−1^ at 35°C) using a mobile phase of water : acetonitrile in the ratio 50 : 50 (v/v).

## Results

### Cytokinin downregulates PSII photochemistry during early stages of dark incubation

To evaluate the effects of BAP and MTU on photosynthetic parameters during different stages of dark incubation, we measured Chl content and the maximum quantum yield of PSII photochemistry (*F*_V_/*F*_M_) at 2, 5, 7, 9, and 12 DAD.

**Fig. 1:**
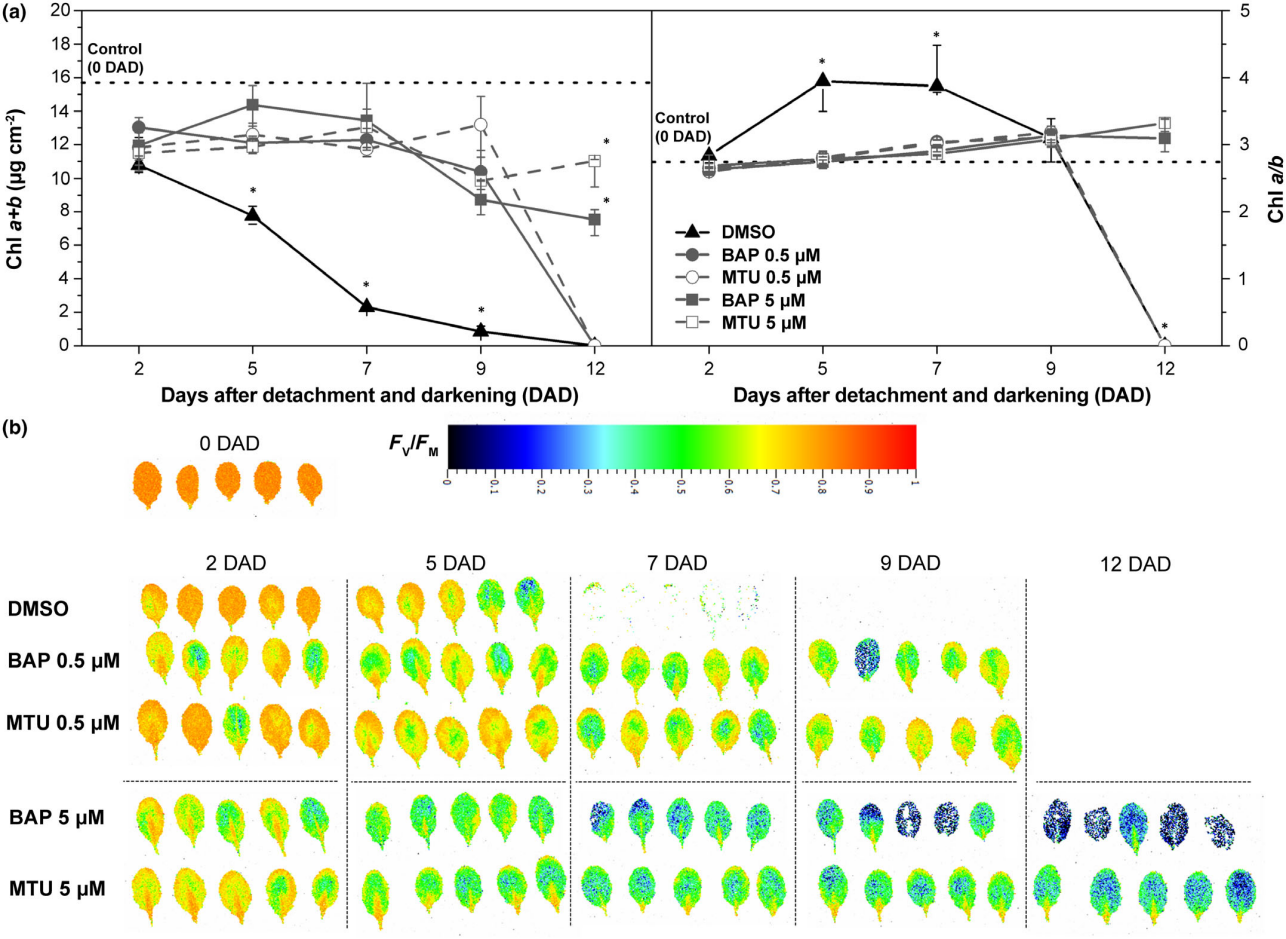
Cytokinins delay the onset of senescence. (a) Chl *a* + *b* content (left) and Chl*a* : Chl*b* ratio (right) and (b) imaging of maximum quantum yield of Photosystem II (PSII) photochemistry (*F*_V_/*F*_M_) in *Arabidopsis thaliana* Columbia‐0 (Col‐0) rosette leaves of 33‐d‐old plants that were detached and treated with mock (0.2% dimethylsulfoxide (DMSO)), benzylaminopurine (BAP) or 1‐(2‐methoxyethyl)‐3‐(1,2,3‐thiadiazol‐5‐yl)urea (MTU) at concentrations of 0.5 or 5 μM, and kept for 2, 5, 7, 9 or 12 d in the dark (days after detachment and darkening, DAD). Freshly detached leaves were used as the control (0 DAD). In (a), medians and quartiles are presented (*n* = 5), and asterisks indicate statistical differences between treatments within a given day (Tukey's test; *P* < 0.05).

As expected, both compounds significantly slowed Chl degradation at both concentrations used (0.5 and 5 μM; Fig. 1a, left). Compared with mock‐treated leaves (0.2% DMSO), Chl content was significantly higher in all CK‐treated leaves from 5 DAD onward, except for 12 DAD, where Chl levels were already undetectable for 0.5 μM treatments and mock controls. At the higher concentration (5 μM), however, both compounds maintained significant amounts of Chl in the leaves even at 12 DAD, with MTU proving more effective than BAP (Fig. 1a, left).

Up to 7 DAD, MTU, and BAP at both concentrations also prevented the increase in the Chl*a* : Chl*b* ratio that typically accompanies dark‐induced senescence in mock‐treated Arabidopsis (Col‐0) leaves (Fig. 1a, right; Kučerová *et al*., 2020). As an elevated Chl*a* : Chl*b* ratio reflects preferential degradation of light‐harvesting complexes (LHCs) relative to the photosynthetic reaction centers, the unchanged ratio measured in the treated leaves indicates that both compounds markedly suppressed this selective LHC degradation.

Clear antisenescence effects of CK on PSII photochemistry became evident from 7 DAD onward, when *F*_V_/*F*_M_ values had already dropped below detection in mock‐treated leaves but remained relatively high (*c*. 0.5) in CK‐treated leaves (Fig. 1b). Interestingly, the higher CK concentration caused a slight decline in PSII photochemistry at 7 DAD and 9 DAD compared with the lower CK concentration, although PSII remained functional up to 12 DAD.

**Fig. 2:**
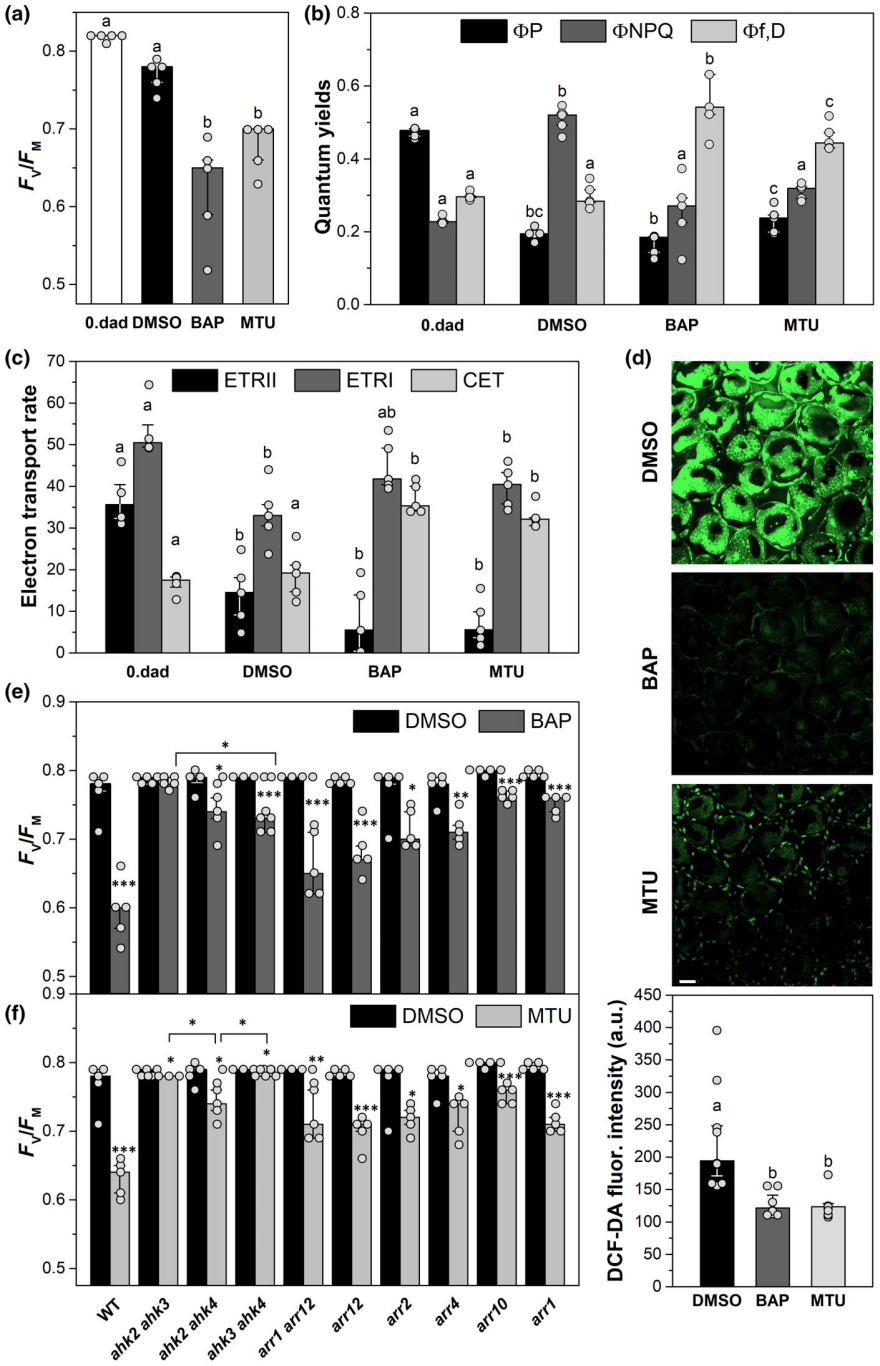
Effects of benzylaminopurine (BAP) and 1‐(2‐methoxyethyl)‐3‐(1,2,3‐thiadiazol‐5‐yl)urea (MTU) treatments on photosynthetic parameters in detached leaves of *Arabidopsis thaliana* at 2 d after darkening. (a) Maximum quantum yield of Photosystem II (PSII) photochemistry (*F*_V_/*F*_M_). (b) Quantum yields of PSII photochemistry (Φ_P_), regulatory nonphotochemical quenching (Φ_NPQ_), and nonregulatory dissipation processes (Φ_f,D_). (c) Electron transport rates through PSII (ETRII), PSI (ETRI), and cyclic electron transport (CET). (d) Reactive oxygen species accumulation visualized using 2′,7′‐dichlorodihydrofluorescein diacetate (DCF‐DA) and quantified from mean green‐fluorescence intensity. (e, f) *F*_V_/*F*_M_ in cytokinin (CK) receptor (*ahk*) double mutants and selected CK response‐regulator (*arr*) mutants. In (a–f), detached leaves of *Arabidopsis thaliana* Columbia‐0 (Col‐0) wild‐type (WT) or selected mutants were incubated with mock (0.2% dimethylsulfoxide (DMSO)), 5 μM BAP, or 5 μM MTU in darkness for 2 d (2 d after detachment and darkening (DAD)). Freshly detached leaves were used as the control (0 DAD). In (d), samples were subsequently illuminated by high light (800 μmol photons m^−2^ s^−1^, 15 min) before imaging. In (a–b), different letters indicate statistically significant differences between treatments for the given parameter (Tukey's test, *P* < 0.05). Medians and quartiles (together with individual data points) are presented (in (a–c): *n* = 4–5; in (d): *n* = 8–10). In (e, f), asterisk brackets denote statistical difference (Tukey's test; *P* < 0.05) between *ahk2 ahk3*, *ahk2 ahk4*, and *ahk3 ahk4* CK double receptor mutants. Asterisks indicate a statistically significant difference (*,P < 0.05; **, P < 0.01; ***, P < 0.001) between mock‐ (DMSO) and CK‐treated leaves (BAP in (e) or MTU in (f)) within the same genotype (Student's *t*‐test). Medians and quartiles are shown (together with individual data points; *n* = 5).

Unexpectedly, at 5 DAD, the lower CK concentration had no noticeable positive effect on PSII photochemistry, while at the higher concentration, *F*_V_/*F*_M_ was even lower than in the mock controls (Fig. 1b). This occurred despite both CKs effectively preserved Chl levels at 5 DAD (Fig. 1a). The CK‐induced suppression of PSII function was even more evident at 2 DAD, where *F*_V_/*F*_M_ in mock‐treated leaves was only slightly reduced compared with control leaves (fresh leaves immediately after detachment, 0 DAD), whereas CK‐treated leaves showed a significant decrease in *F*_V_/*F*_M_ (Figs 1b, 2a).

### Effects of BAP and MTU on photosynthetic parameters

To better understand this unusual CK effect on primary photosynthetic processes during the early stages of incubation in darkness, here referred to as ‘prolonged darkness’, we measured photosynthetic parameters after 2 DAD (which represents three times the standard night phase and corresponds to a stage before the onset of dark‐induced senescence), following 10 min of leaf illumination (i.e. in the light‐adapted state). The rate of utilization and/or dissipation of absorbed light energy was evaluated by quantifying Φ_P_ (the effective quantum yield of PSII photochemistry in the light‐adapted state), Φ_NPQ_ (quantum yield of regulatory nonphotochemical quenching), and Φ_f,D_ (quantum yield of constitutive nonregulatory dissipation processes) (Lazár, 2015).

The quantum yields were measured under approximately doubled light intensity compared with standard growth conditions to evaluate the ability of leaves to utilize increased light energy. At 2 DAD, Φ_P_ decreased to a similar extent in both mock‐ and CK‐treated leaves (Fig. 2b). Thus, in contrast to PSII photochemistry in the dark (i.e. *F*_V_/*F*_M_), none of the CKs stimulated the downregulation of PSII photochemistry in the light‐adapted state. However, although the decrease in Φ_P_ was similar in the mock‐ and CK‐treated leaves, the underlying causes were different: In mock‐treated leaves, the reduction in Φ_P_ was accompanied by an increase in Φ_NPQ_, whereas in CK‐treated leaves, Φ_f,D_ increased (Fig. 2b). Thus, the decrease in Φ_P_ in mock‐treated leaves was caused by stimulation of light‐induced nonphotochemical quenching, while BAP and MTU promoted light‐independent constitutive quenching processes that quenched *F*_M_, resulting in the *F*_V_/*F*_M_ decrease (Fig. 2a). The nature of these quenching processes is unclear, but it does not appear to reflect photodamage, which typically causes a decrease in *F*_V_/*F*_M_, or any other type of PSII damage or degradation. Instead, we assume that only a functional downregulation of PSII photochemistry occurred in the CK‐treated leaves. This downregulation was fully reversible: Upon returning the leaves to a normal photoperiodic regime, the decrease in *F*_V_/*F*_M_ in CK‐treated leaves progressively disappeared, and their *F*_V_/*F*_M_ values became indistinguishable from those of mock‐treated leaves (Fig. S1).

To assess how the CK treatment affects primary photosynthetic reactions at even higher light supply, we measured the electron transport rates through PSII (ETRII) and PSI (ETRI) under a light intensity of 800 μmol photons m^−2^ s^−1^. Compared with control leaves, ETRII decreased in both mock‐ and CK‐treated leaves, with the reduction being slightly (but not significantly) greater in CK‐treated leaves (Fig. 2c). By contrast, the decline in ETRI was less pronounced in CK‐treated leaves than in mock‐treated leaves, although this difference was also not statistically significant. Notably, CET around PSI was significantly stimulated in CK‐treated leaves compared with both mock‐treated and control leaves (Fig. 2c). CET is considered a protective mechanism against the accumulation of ROS and the associated photo‐oxidative damage to the photosynthetic apparatus caused by excess excitation energy (Yamori & Shikanai, 2016). Thus, the CK treatment should reduce ROS accumulation in the leaves exposed to high light. Indeed, ROS levels after high‐light (800 μmol photons m^−2^ s^−1^) exposure were significantly reduced in the CK‐treated leaves (Fig. 2d).

Overall, both compounds in darkened leaves prevented Chl and LHC degradation, while activating light‐independent quenching processes that downregulated PSII photochemistry. In the light‐adapted state, PSII photochemistry in the CK‐treated leaves was comparable to that in mock‐treated leaves. Further, PSI function, including CET, was enhanced under high‐light conditions, coincident with the reduced ROS accumulation. These findings lead us to propose that the CK‐induced downregulation of PSII photochemistry may serve as a protective mechanism, shielding the photosynthetic apparatus from overexcitation and photo‐oxidative damage during the transition from dark‐ to light‐adapted states.

To determine whether both compounds mediate the downregulation of PSII photochemistry via the CK phosphorelay pathway, we examined the effects of CK treatment in double‐knockout mutants for the CK receptor genes *AHK2*, *AHK3*, and *CRE1/AHK4* (Riefler *et al*., 2006). As shown in Fig. 2(e,f), different degrees of suppression in PSII photochemistry were observed. Among the receptor mutants, the *ahk2 ahk3* double mutant almost completely prevented the decline in *F*_V_/*F*_M_ (for both BAP and MTU) compared with mock‐treated controls. Similarly, in MTU‐treated leaves, the *ahk3 ahk4* double mutant displayed comparable behavior. This suggests that the CK‐induced downregulation of PSII photochemistry is primarily mediated by the AHK3 and AHK2 receptors in response to BAP, while the AHK3 receptor appears to be the main target in MTU‐treated leaves.

By contrast, deficiencies in specific *ARR* single or double mutants only weakly attenuated the CK effect (Fig. 2e,f). However, ARR1, ARR10, and possibly the A‐type regulator ARR4 appear to contribute to some extent. Interestingly, the *arr1* deficiency provoked a stronger effect than the *arr1 arr12* double mutant, warranting further investigation. This could suggest opposing roles for ARR1 and ARR12 in regulating this process. ARR1 is known to inhibit shoot regeneration and callus formation, while ARR12 may promote these processes under certain conditions (Liu *et al*., 2020).

### Cytokinin modulates gene networks involved in hormone signaling and light perception in dark‐incubated leaves

To elucidate the mechanism underlying the CK action during prolonged darkness, we performed a genome‐wide transcriptomic analysis of CK‐ and mock‐treated (DMSO control) Arabidopsis leaves at 48 h (2 DAD). In addition, this analysis was also performed 6 h after leaf detachment and CK treatment to capture more immediate responses to BAP and MTU applications. Principal component analysis (PCA) revealed distinct clusters of biological replicates at both time points (Fig. S2).

**Fig. 3:**
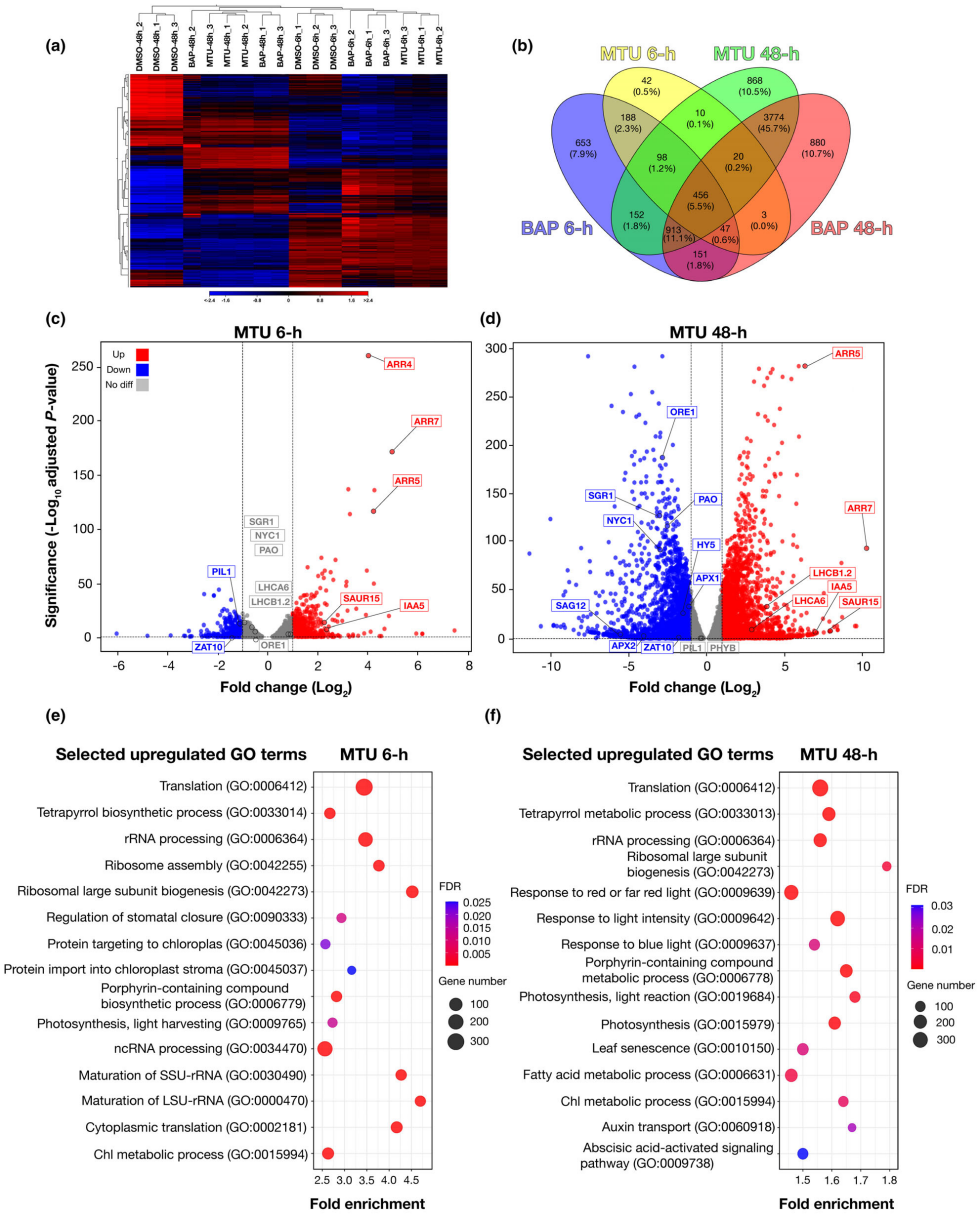
Transcriptomic analysis of dark‐incubated *Arabidopsis thaliana* leaves treated with cytokinins (CK) reveals upregulation of photosynthesis‐ and light‐sensing‐related pathways. Illumina NovaSeq next‐generation sequencing was used to compare transcriptomic profiles of mock‐treated samples with those treated with 5 μM benzylaminopurine (BAP) or 5 μM 1‐(2‐methoxyethyl)‐3‐(1,2,3‐thiadiazol‐5‐yl)urea (MTU). (a) Heatmap illustrating the clustering of differentially expressed genes (DEGs) across the samples. Blue indicates downregulated genes, and red indicates upregulated genes, with color saturation reflecting the magnitude of differential expression. (b) Venn diagram comparing DEGs in *Arabidopsis thaliana* Columbia‐0 (Col‐0) leaves after short‐term (6 h) and long‐term (48 h) treatment with BAP or MTU. Both upregulated and downregulated DEGs with a |log_2_(fold change)| > 1 are included. (c, d) Volcano plots showing next‐generation sequencing (NGS) results for samples treated with MTU, for 6 or 48 h, respectively. Selected genes of CK signaling (*ARR4*, *ARR5*, and *ARR7*), auxin signaling (*IAA5* and *SAUR15*), Photosystem II (PSII)/PSI (*LHCB1.2* and *LHCA6*), photomorphogenesis/shade (*PHYB*, *HY5* and *PIL1*), Chl degradation/senescence (*PAO*, *NYC1*, *SGR1*, *ORE1* and *SAG12*) and reactive oxygen species markers (*APX1*, *ZAT10*, and *ZAT12*) are boxed. (e, f) Gene Ontology (GO) enrichment analysis performed using the PANTHER database, showing highly enriched GO terms after short‐term (6 h) and long‐term (48 h) MTU treatments, respectively. The *x*‐axis displays a fold enrichment, which indicates how strongly a GO term is overrepresented in the input gene list compared with its expected frequency in the reference genome. The bubble size represents the number of DEGs related to the depicted GO term, and the bubble hue indicates the false discovery rate (FDR; cutoff < 0.05). Overrepresentation was assessed using Fisher's exact test with Benjamini–Hochberg FDR correction.

The 48‐h MTU treatment resulted in extensive transcriptional reprogramming, with 4997 differentially expressed genes (DEGs) identified after 6 h (2588 upregulated and 2409 downregulated) and 13 939 DEGs after 48 h (6844 upregulated and 7095 downregulated), all meeting an adjusted *P*‐value of < 0.05. A comparable number of DEGs were observed with the canonical CK BAP, as demonstrated by hierarchical clustering (Fig. 3a). DEGs from both CK treatments showed distinct clustering of upregulated and downregulated genes, with greater changes observed after the 48‐h treatment. All treatments regulated a common set of 456 genes with a |log_2_(fold change)| > 1 (Fig. 3b), capturing CK responses preceding the onset of dark‐induced senescence. Of these, 297 genes were upregulated, and 159 were downregulated (Tables 1, S2).

**Table 1:** Overview of differentially expressed genes (DEGs) regulated by benzylaminopurine (BAP) or 1‐(2‐methoxyethyl)‐3‐(1,2,3‐thiadiazol‐5‐yl)urea (MTU) treatment in detached and darkened leaves of *Arabidopsis thaliana*.

AGI no.	BAP treatment (Log_2_ FC)		MTU treatment (Log_2_ FC)		Gene description
	6 h	48 h	6 h	48 h	
*Upregulated*					
At1g19050	5.80	10.57	4.97	10.31	ARR7, two‐component response regulator 7
At4g15660	1.42	9.40	1.10	9.56	GRXS8, a member of the CC‐type glutaredoxin (ROXY) family
At4g38850	2.79	7.84	2.28	7.94	SAUR15, ARABIDOPSIS THALIANA SMALL AUXIN UPREGULATED 15
At2g30540	4.40	7.88	3.96	7.55	GRXS9, a member of the CC‐type glutaredoxin (ROXY) family
At5g56970	6.18	7.65	3.41	4.39	CKX3, cytokinin oxidase/dehydrogenase 3
At1g15580	3.44	6.52	2.36	6.85	IAA5, INDOLE‐3‐ACETIC ACID INDUCIBLE 5
At1g52830		5.39		7.00	IAA6 / SHY1, short hypocotyl 1
At3g57040	3.61	6.34	2.34	6.33	ARR9, two‐component response regulator 9
At1g29490	2.97	5.20	1.46	5.99	SAUR68, SMALL AUXIN UPREGULATED 68
At3g48100	5.24	6.11	4.23	5.94	ARR5, two‐component response regulator 5
At1g10460	9.58	7.28	7.46	5.89	GLP7, GERMIN‐LIKE PROTEIN 7
At2g01830	4.81	6.22	4.24	5.84	CRE1/AHK4, cytokinin‐binding histidine kinase
At1g10470	4.47	5.77	4.03	5.71	ARR4, two‐component response regulator 4
At1g29430	2.13	5.20	1.72	5.61	SAUR62, SMALL AUXIN UPREGULATED RNA 62
At5g39860	3.96	4.85	3.54	5.61	PRE1, PACLOBUTRAZOL RESISTANCE 1
At5g05860	2.93	5.86	2.71	5.52	UGT76C2, UDP‐GLUCOSYL TRANSFERASE 76C2
At5g20740	2.87	5.48	2.08	5.48	PMEI3, PECTIN METHYLESTERASE INHIBITOR 3
At3g54720	1.47	5.49	1.30	5.47	COP2, CONSTITUTIVE MORPHOGENESIS 2
At1g04240	1.23	2.96	0.87	3.32	IAA3 / SHY2, short hypocotyl 2
At1g18400	1.26	3.23	0.81	2.92	BEE1, brassinosteroid signaling component
*Downregulated*					
At2g44990	−5.39	−13.42	−6.01	−9.17	CCD7, CAROTENOID CLEAVAGE DIOXYGENASE 7
At1g09240	−3.18	−11.38	−1.76	−10.01	NAS3, NICOTIANAMINE SYNTHASE 3
At4g32810	−2.06	−10.85	−1.64	−11.36	CCD8, CAROTENOID CLEAVAGE DIOXYGENASE 8
At3g08860	−2.54	−10.43	−1.96	−9.06	PYD4, PYRIMIDINE 4, predicted beta‐alanine aminotransferase activity
At3g03480	−4.35	−11.75	−2.64	−7.48	CHAT, ACETYL COA:(Z)‐3‐HEXEN‐1‐OL ACETYLTRANSFERASE
At1g48260	−3.53	−6.88	−1.39	−7.59	CIPK17, CBL‐INTERACTING PROTEIN KINASE 17
At5g63850	−2.30	−5.16	−1.32	−5.99	AAP4, AMINO ACID PERMEASE 4
At4g01430	−4.94	−7.64	−3.13	−5.96	UMAMIT29, USUALLY MULTIPLE ACIDS MOVE IN AND OUT TRASPORTER2
At3g10320	−6.96	−6.90	−2.53	−5.83	MUCI21, MUCILAGE‐RELATED 21
At3g14280	−2.73	−6.33	−1.20	−5.51	LL‐Diaminopimelate aminotransferase
At5g43180	−2.81	−6.08	−2.12	−5.29	Transmembrane protein
At2g32660	−2.95	−5.62	−1.74	−5.26	RLP22, RECEPTOR LIKE PROTEIN 22
At2g34080	−3.06	−7.53	−1.45	−5.26	Cysteine proteinases superfamily protein
At1g01453	−4.73	−7.22	−2.03	−5.26	Late embryogenesis abundant hydroxyproline‐rich glycoprotein family protein
At4g11910	−2.23	−5.39	−1.84	−5.19	NYE2, NONYELLOWING 2
At1g34060	−2.23	−5.59	−1.45	−5.19	TAR4, TRYPTOPHAN AMINOTRANSFERASE RELATED 4
At4g18980	−2.15	−5.01	−1.54	−5.06	AtS40‐3, encodes a nuclear‐targeted protein AtS40‐3 that modulates senescence‐associated gene expression
At1g69500	−2.78	−5.35	−2.01	−4.97	CYP704B1, CYTOCHROME P450, FAMILY 704

The table presents a selection of the most strongly upregulated and downregulated genes (adjusted *P*‐value < 0.05) with the highest absolute log_2_ fold change (log_2_FC) after 6 or 48 h of dark incubation of detached *Arabidopsis thaliana* wild‐type leaves in the presence of 5 μM BAP or MTU.

Gene Ontology (GO) overrepresentation analysis of the upregulated genes revealed enrichment in categories related to ribosome function and biogenesis, as well as CK and other plant hormone signaling pathways. Notably, we observed the upregulation of classical type‐A *ARRs* and other CK‐responsive genes (Brenner *et al*., 2012; Brenner & Schmülling, 2015), several members of the *Small Auxin Up‐Regulated RNA (SAUR)* family, and some photomorphogenesis‐related genes (*PRE1*, *COP2*; Fig. 3c,d; Table 1). Conversely, among the commonly downregulated genes, we noted suppressed several metabolic pathways, such as the glycerolipid pathway and amino acid biosynthesis (arginine and proline). Additionally, *NYE2 – a* positive regulator of Chl degradation (Sakuraba *et al*., 2014; Wu *et al*., 2016) – was significantly downregulated in both CK treatments, along with *AtS40‐3*, a master regulator of leaf senescence responsive also to dark treatment or pathogen attack (Fischer‐Kilbienski *et al*., 2010; Table 1). Consistent with this, *SAG12*, a hallmark senescence‐associated gene, and *NYC1*, encoding a reductase required for Chl and LHC degradation, were also downregulated following MTU treatment (Fig. 3d). These findings confirm that both CK treatments, up to the 2 DAD time point, actively prevent the onset of leaf senescence and Chl degradation during prolonged darkness.

Further transcriptional reprogramming induced by both CK treatments was highlighted through GO term overrepresentation analysis using the AgriGO database (Fig. S3). Both BAP and MTU activated the CK pathway and auxin signaling during prolonged darkness to a similar extent (Table 1), but we also noted some interesting differences between the two compounds. For instance, BAP induced stronger upregulation of *CKX3*, encoding the CKX enzyme, whereas MTU had a greater impact on genes associated with photosynthesis, organ growth regulation, and light perception. GO enrichment analysis using the PANTHER database (Mi *et al*., 2019) reinforced these findings, identifying significant enrichment of categories, such as ‘response to red and far‐red light’, ‘response to light intensity’, ‘photosynthesis’, and ‘chlorophyll metabolic process’ in MTU‐treated samples (Fig. 3e,f; Table S3).

In line with previous reports, the two CKs also showed distinct effects on root development in Arabidopsis seedlings. While exogenous application of BAP markedly inhibited primary root elongation and reduced lateral root formation, MTU effects were much milder (Fig. S4A–C). These morphological differences were coincident with a significantly weaker CK signaling response to MTU (Table S4), notably in the stele region, as demonstrated in the *pTCSv2::3XVENUS* assay and also supported by qRT‐PCR analysis of type‐A *ARR* transcripts, suggesting different kinetics in MTU‐mediated signaling compared with BAP (Fig. S4D–F).

### Correlation between proteomic and transcriptomic analyses confirms regulation of photosynthesis and light‐related processes in darkened leaves

**Fig. 4:**
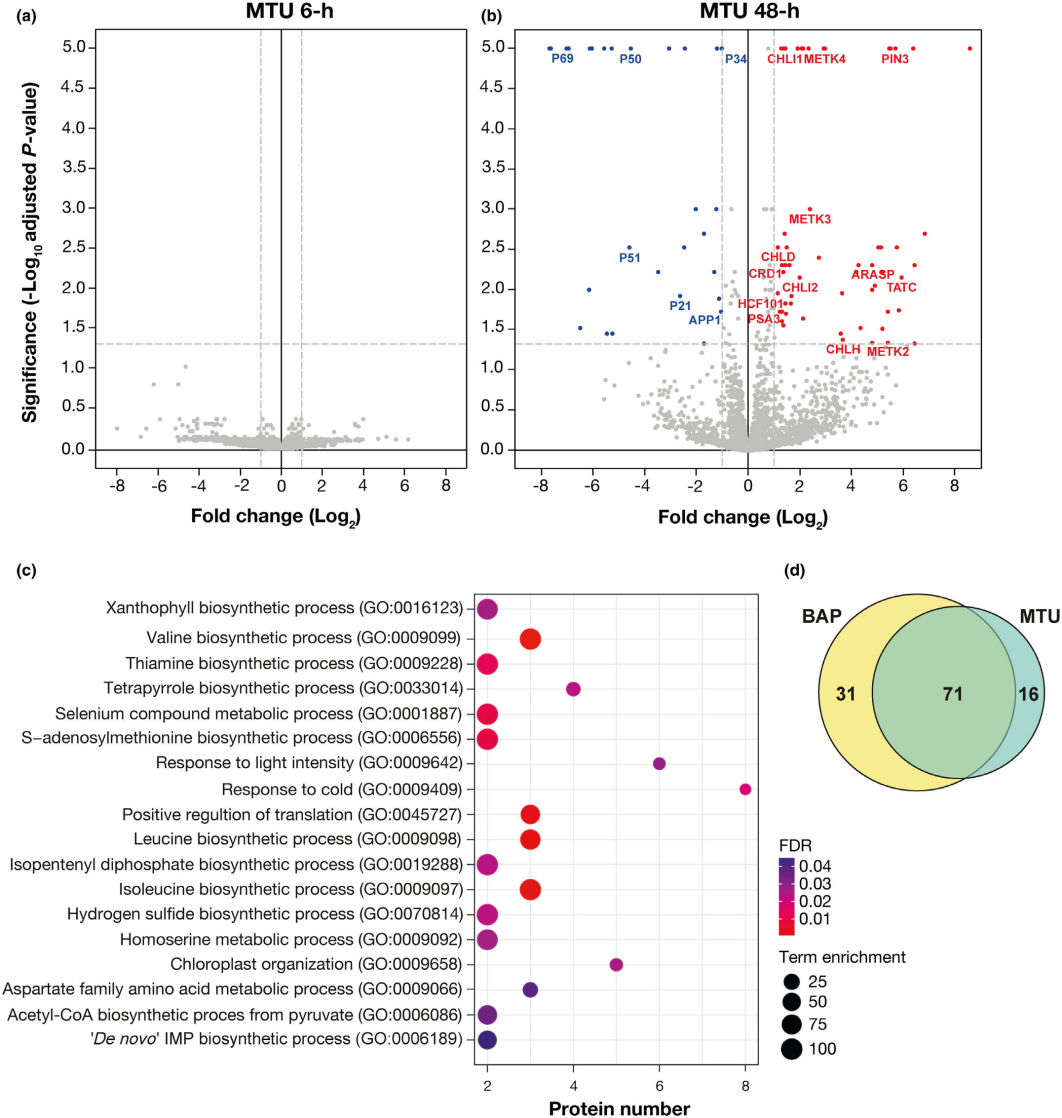
Proteomic analysis of *Arabidopsis thaliana* leaves treated with cytokinins (CKs). (a, b) Volcano plots showing proteins with significant differences in abundance after the indicated CK treatment. Detached *Arabidopsis thaliana* Columbia‐0 (Col‐0) leaves were incubated in mock solution (0.2% dimethylsulfoxide (DMSO)) or 5 μM 1‐(2‐methoxyethyl)‐3‐(1,2,3‐thiadiazol‐5‐yl)urea (MTU) in the dark for 6 h (a) or 48 h (b). −Log_10_(Benjamini–Hochberg adjusted *P*‐values) and Log_2_(Fold change) are shown on the *y*‐ and *x*‐axis, respectively. Nonaxial lines denote cutoffs for statistically significant changes in adjusted *P*‐value (< 0.05) and fold change (> 2). For visualization purposes, proteins with *P*‐values of 0 were replaced with 1 × 10^−5^ to allow their display in the plot. Downregulated proteins are shown in blue and upregulated proteins in red. (c) Significantly enriched Gene Ontology (GO) terms connected with the differentially abundant proteins for MTU treatment. The *x*‐axis displays the number of proteins related to the depicted GO term, the bubble size represents the term enrichment (relative overrepresentation of the given GO category compared with the reference proteome), and the bubble hue symbolizes the respective false discovery rate (FDR) value (applied cutoff < 0.05). (d) A Venn diagram depicting the overlap between proteins that showed significantly different abundance in response to 5 μM benzylaminopurine (BAP) (yellow) or 5 μM MTU (teal) after 48 h.

To complement the transcriptomic results, we performed label‐free quantitative proteomic analysis of detached Arabidopsis leaves after MTU and BAP treatments (Figs 4a,b, S5A,B). A total of 3052 proteins were identified (Table S5), and 118 displayed significantly altered abundance at 2 DAD (adjusted *P*‐value <0.05; |log_2_FC| > 1), with *c*. 60% overlap between treatments, indicating similar modes of action (Fig. 4d). Of these, 59 and 68 proteins were upregulated and 28 and 34 were downregulated in MTU‐ and BAP‐treated samples, respectively (Tables S6, S7). GO enrichment analysis revealed significant overrepresentation of processes associated with photosynthesis, chloroplast organization, translation, and amino acid metabolism (Figs 4c, S5C), partially overlapping with MTU‐driven transcriptomic trends (Fig. 3e,f).

Closer inspection of the protein dataset showed that both CKs promoted the accumulation of important enzymes involved in Chl biosynthesis (e.g. CRD1, CHLI1, CHLI2, CHLH, and CHLD) and proteins linked to photosystem assembly/stability and overall chloroplast organization (e.g. HCF101, PSA3, TIC56, TATC, and ARASP).

Among the upregulated proteins, several methionine adenosyltransferases (METK2, METK3, and METK4; Fig. 4b) were detected, which catalyze the formation of S‐adenosylmethionine (SAM), a central methyl donor in multiple biosynthetic and signaling processes. This supports previous reports indicating that SAM metabolism is sensitive to CK signaling and may contribute to redox regulation and methylation‐dependent control of chloroplast gene expression (Zavaleta‐Mancera *et al*., 2007; Cortleven & Schmülling, 2015).

Another enriched group comprised enzymes of branched‐chain amino acid (BCAA) metabolism (Table S8), such as 2‐isopropylmalate synthase 1 (MAM‐like 4) and branched‐chain‐amino acid aminotransferases 2 and 3 (BCAT2/3). Their increased abundance suggests metabolic readjustment to sustain anabolic precursors and maintain minimal ATP production under prolonged darkness.

By contrast, proteins associated with ROS metabolism, notably class III peroxidases (P21, P34, P50, P51, and P69), were consistently downregulated in both treatments (Fig. 4b). This finding aligns with confocal ROS imaging, where CK‐treated leaves showed reduced ROS accumulation (Fig. 2d), supporting the view that under prolonged darkness, CKs modulate antioxidant networks to prevent excessive oxidative stress. Finally, the cytosolic glutamine synthetase GLN1;1, a key enzyme in nitrogen assimilation and stress responses (Ishiyama *et al*., 2004), was found to be downregulated, possibly reflecting a shift away from nitrogen remobilization typical of early senescence (Li *et al*., 2006; Tables S6, S7).

**Fig. 5:**
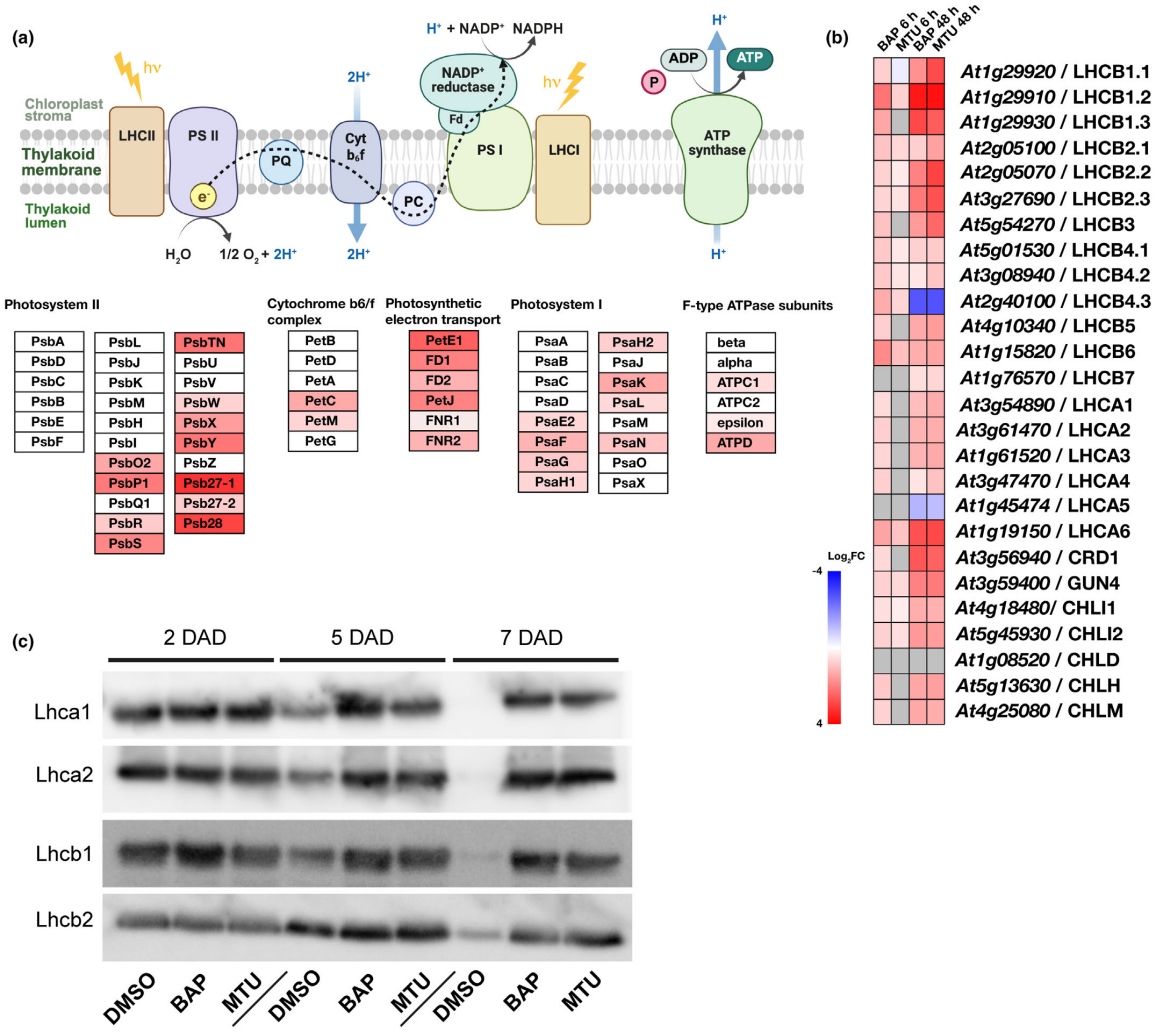
Interaction of the cytokinin (CK) pathway with photosynthesis and light sensing. (a, b) Schematic summary of differentially expressed genes (DEGs) from RNA‐seq associated with photosynthesis and light‐harvesting in detached and darkened *Arabidopsis thaliana* leaves following CK treatment. Solid arrows indicate the direction of proton flow or product formation, whereas the dashed arrow indicates the electron flow through the photosynthetic electron transport chain. Upregulated DEGs are shown in red, and downregulated DEGs are shown in blue, color‐coded according to the same scale as in panel b. Created with BioRender (https://BioRender.com/w5kzmbn). (b) Heatmap of transcript abundance for selected DEGs in response to benzylaminopurine (BAP) or 1‐(2‐methoxyethyl)‐3‐(1,2,3‐thiadiazol‐5‐yl)urea (MTU) after 6 and 48 h of treatment in darkened, detached leaves. Expression values are color‐coded according to the log₂ scale shown. (c) Immunoblot analysis of wild‐type leaf protein extracts probed with antibodies against light‐harvesting complex proteins: Lhca1, Lhca2, Lhcb1, and Lhcb2. Detached leaves were treated with mock (0.2% dimethylsulfoxide (DMSO)), 5 μM BAP or 5 μM MTU and kept in darkness for 2, 5, or 7 d (days after detachment and darkening (DAD)).

Integration of the proteomic and transcriptomic datasets consistently highlighted GO terms associated with photosynthesis, particularly light harvesting, light and dark reactions, and assembly of PSII and PSI (Tables S3, S6, S7). A detailed analysis of DEGs associated with the photosystem assembly revealed a significant upregulation of most antenna proteins, particularly in PSII (Fig. 5a,b). Both BAP and MTU triggered the upregulation of these genes (including *LHCA6*), with similar magnitudes, except for *LHCB4.3* and *LHCA5*, which were strongly downregulated (Fig. 5b). At the protein level, immunoblotting with anti‐Lhca1/2 and anti‐Lhcb1/2 antibodies corroborated these trends; however, with a temporal lag. The preservation of major LHCI and LHCII components in CK‐treated vs mock‐treated leaves was clearly visible at 5 DAD and especially 7 DAD (Fig. 5c).

Intriguingly, both CKs also induced a group of genes typically associated with red‐/FR light signaling and photomorphogenesis (Table 1), including *SHY1* and *SHY2* (Kim *et al*., 1996, 1998), along with *BEE1*, which links brassinosteroid signaling to CK responses (Gautrat *et al*., 2024). Together, these transcriptional changes point to an involvement of phyB, the red/FR photoreceptor, in integrating CK perception with light signaling. This is consistent with recent models in which phyB activity modulates CK output via repression of A‐type *ARRs* by PIF factors (Gautrat *et al*., 2024).

At 2 DAD, qPCR assays confirmed contrasting expression patterns of *PHYA* and *PHYB* in leaves treated with CKs, in agreement with our transcriptomic results (Figs S6, S7). *PHYB* transcript levels were reduced by CK treatment by > 50%, while *PHYA* expression was significantly upregulated by MTU treatment. In addition, both CKs upregulated genes encoding components of the chloroplastic NDH complex (*NDHN* and *NDHM*) together with *CRR1*, which is implicated in NDH complex stabilization (Shimizu & Shikanai, 2007). This is in line with the observed stimulation of CET under CK treatment (Fig. 2c), as the NDH complex is involved in CET (Yamori & Shikanai, 2016).

Overall, these data indicate that during prolonged darkness CKs reprogram leaves at multiple levels: (1) they stabilize chloroplast structure and biosynthetic performance; (2) they limit ROS accumulation and suppress early senescence‐like nutrient remobilization; (3) they modulate amino acid metabolism toward a metabolically restrained, low‐turnover state; and (4) they reshape light‐signaling networks, including phyB‐linked transcriptional outputs. Notably, MTU elicited a slower but ultimately a strong transcriptional response comparable to BAP, particularly at 2 DAD, suggesting differences in receptor engagement and/or signaling kinetics between these two CK‐related compounds.

### Cytokinin‐mediated transcriptional reprogramming under prolonged darkness depends on phyB

Based on our omics results pointing to integration of CK and light‐signaling modules, we next asked whether the CK‐induced transcriptional changes themselves depend on phyB. To this end, we quantified selected transcript abundance by qPCR in WT and *phyB* leaves at 2 DAD.

**Fig. 6:**
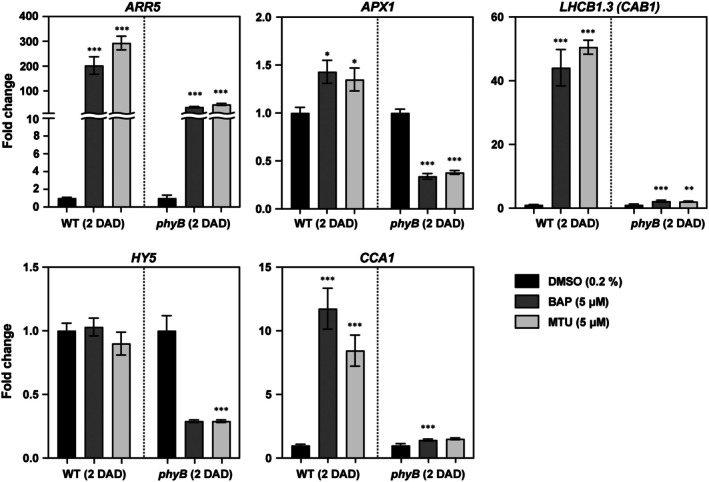
Crosstalk of cytokinin and light signaling in *Arabidopsis thaliana* wild‐type (WT) and *phyB* mutant plants. Reverse transcription quantitative polymerase chain reaction (RT‐qPCR) analysis of *ARR5*, *APX1*, *CAB1*, *HY5*, and *CCA1* transcript levels in detached leaves of 6‐wk‐old *Arabidopsis thaliana* Columbia‐0 (Col‐0) (WT) and *phyB* mutant line (SALK_022035C) after 2 d in darkness (2 d after detachment and darkening (DAD)). Leaves were treated with mock (0.2% dimethylsulfoxide (DMSO)), 5 μM benzylaminopurine (BAP), or 5 μM 1‐(2‐methoxyethyl)‐3‐(1,2,3‐thiadiazol‐5‐yl)urea (MTU). Three independent biological experiments were performed. For each biological replicate, at least 20 leaves collected from at least five individual plants per treatment were pooled for RNA extraction. Relative transcript abundance was calculated using the ΔΔ*C*_t_ method, normalized to reference genes, and expressed as fold change relative to the mock‐treated sample of the corresponding genotype. Bars show mean ± SE. Statistical significance of differences between mock‐ and CK‐treated samples within each genotype was assessed by Student's *t*‐test (*, *P* < 0.05; **, *P* < 0.01; ***, *P* < 0.001).

Although the absolute fold changes measured by qPCR were often larger than those obtained from RNA‐seq, reflecting the higher dynamic range and gene‐specific normalization of qPCR, the direction of the response was generally consistent between the two methods. By qPCR, the A‐type response regulator *ARR5* was induced by BAP and MTU by more than two orders of magnitude in WT leaves after 2 d of darkness, whereas its induction in *phyB* was strongly reduced (Fig. 6). Similarly, CK strongly upregulated *LHCB1.3* (a major PSII light‐harvesting subunit) and *CCA1* (a chloroplast‐associated transcriptional regulator) in WT, but this response was largely lost in *phyB*. By contrast, a mild CK‐mediated accumulation of *APX1*, a ROS‐scavenging ascorbate peroxidase, was observed in WT but attenuated in *phyB*. However, in our transcriptomic data, we observed modest but statistically significant downregulation of *APX1* transcript levels, consistent with the downregulation of two other ROS‐associated marker genes, *ZAT10* and *ZAT12*. Furthermore, both qPCR and RNA‐seq results confirmed slightly reduced *HY5* transcript abundance in WT in response to MTU treatment (Fig. 3d), and this decrease was even more pronounced in *phyB* (Fig. 6). HY5 activity is directly linked to Chl biosynthesis and, via its interaction with GLK1/2 module, also contributes to the regulation of PSII/PSI‐associated genes; thus, its reduced abundance in *phyB* may contribute to the diminished support of photosynthesis‐related functions compared with CK‐treated WT leaves.

Taken together, these data indicate that CK treatments in darkened leaves activate a coordinated phyB‐dependent transcriptional module that maintains photosynthetic apparatus (*LHCB1.3*, *CCA1*) and also reconfigures ROS‐handling (*APX1*). Crucially, the amplitude of all these modules is diminished in the *phyB* mutant. This demonstrates that phyB is required for the full CK‐responsive transcriptional reprogramming of darkened leaves.

### Cytokinin–phyB crosstalk controls the downregulation of Photosystem II photochemistry

**Fig. 7:**
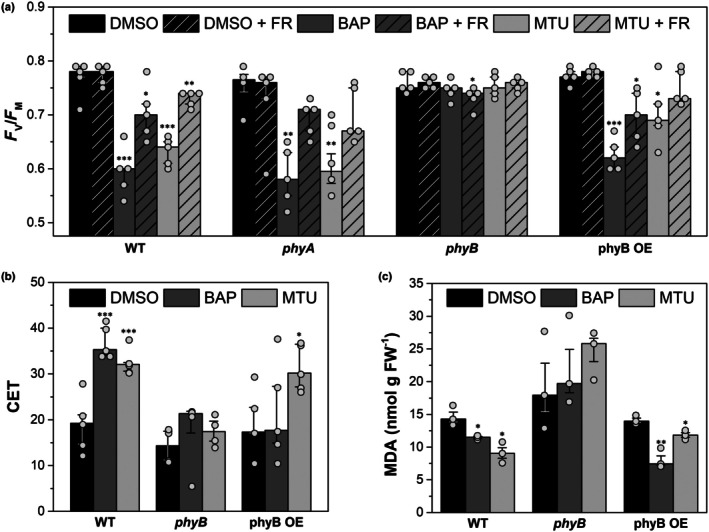
Effects of cytokinin treatments on photosynthetic parameters and lipid peroxidation in *Arabidopsis thaliana* phytochrome mutants. (a) Maximum quantum yield of Photosystem II (PSII) photochemistry (*F*_V_/*F*_M_), (b) cyclic electron transport (CET), and (c) malondialdehyde (MDA) content, in detached leaves of *Arabidopsis thaliana* genotypes: Columbia‐0 (Col‐0) (wild‐type, WT), phytochrome‐deficient mutants *phyA* (SALK_014575C) and *phyB* (SALK_022035C), and phytochrome B‐overexpressing line (*35S::PHYB:GFP*, phyB OE). For (a), leaves were detached and immediately incubated in mock (dimethylsulfoxide (DMSO)), 5 μM BAP, or 5 μM 1‐(2‐methoxyethyl)‐3‐(1,2,3‐thiadiazol‐5‐yl)urea (MTU) for 2 d in darkness (2 d after detachment and darkening (DAD)). +FR variant: leaves were detached, dark‐adapted, exposed to far‐red light (20 μmol photons m^−2^ s^−1^) for 15 min while floating on deionized water, then transferred to BAP/MTU solutions and kept in darkness for 2 d. For (b, c), leaves were detached and incubated with mock/BAP/MTU in darkness; for (c), leaves were subsequently exposed to high light (800 μmol photons m^−2^ s^−1^) for 15 min before MDA quantification. Bars show medians and quartiles (*n* = 3–5; individual data points are shown). Asterisks indicate significant differences vs mock within genotype (Student's *t*‐test; *P* < 0.05).

Additional physiological analyses showed that the CK‐induced decline in *F*_V_/*F*_M_, a key measure of efficiency of PSII photochemistry, was largely unchanged in the CK‐treated leaves of the *phyA* mutant compared with WT (Fig. 7a). By contrast, this decrease was completely abolished in the *phyB* mutant (Fig. 7a). To complement the *phyB* mutant analysis, we also exposed leaves of different genotypes to FR light, which accelerates phyB inactivation (dark reversion). Importantly, leaves were subjected to the FR treatment before being transferred to CK solutions, that is before the onset of CK signaling in darkened leaves. Following this FR pretreatment (for spectrum, see Fig. S8), the CK‐induced decrease in *F*_V_/*F*_M_ was attenuated, including WT and *phyA* leaves (Fig. 7a; +FR variant).

To access the effect of phyB constitutive overexpression (phyB OE), we used a *35S::PHYB:GFP* line (Rausenberger *et al*., 2010). Similar to WT leaves, phyB OE showed a drop in *F*_V_/*F*_M_, particularly in response to BAP treatment. These results altogether suggest that phyB is essential for the CK‐induced downregulation of PSII photochemistry.

In addition, phyB appears to play a role in the CK‐mediated stimulation of CET, as this stimulation was significantly diminished in the *phyB* mutant compared with WT (Fig. 7b). While CET increased by 60–80% in WT, it only increased by 25–50% in *phyB*, indicating that phyB enhances, but is not strictly required for CET stimulation.

The suggested protective role of CET against photo‐oxidative damage was confirmed by the suppression of lipid peroxidation after high‐light treatment in the CK‐treated WT and phyB OE leaves (Fig. 7c). However, this CK‐protection of lipids was not observed in *phyB*, despite the mild CET stimulation. Thus, the CET stimulation in *phyB* might be insufficient to protect lipids from oxidative damage. By contrast, in the phyB overexpressing line, the decrease in MDA was even more pronounced, particularly after BAP treatment, suggesting that elevated phyB levels enhance CK‐dependent protection against oxidative damage (Fig. 7c).

These findings support a model in which phyB positively contributes to the CK‐mediated stimulation of CET and to the protective reduction in lipid peroxidation, with both processes being attenuated in the absence of phyB and partly strengthened when phyB is overexpressed.

### Cytokinin–phyB‐mediated downregulation of PSII also occurs in darkened intact plants

**Fig. 8:**
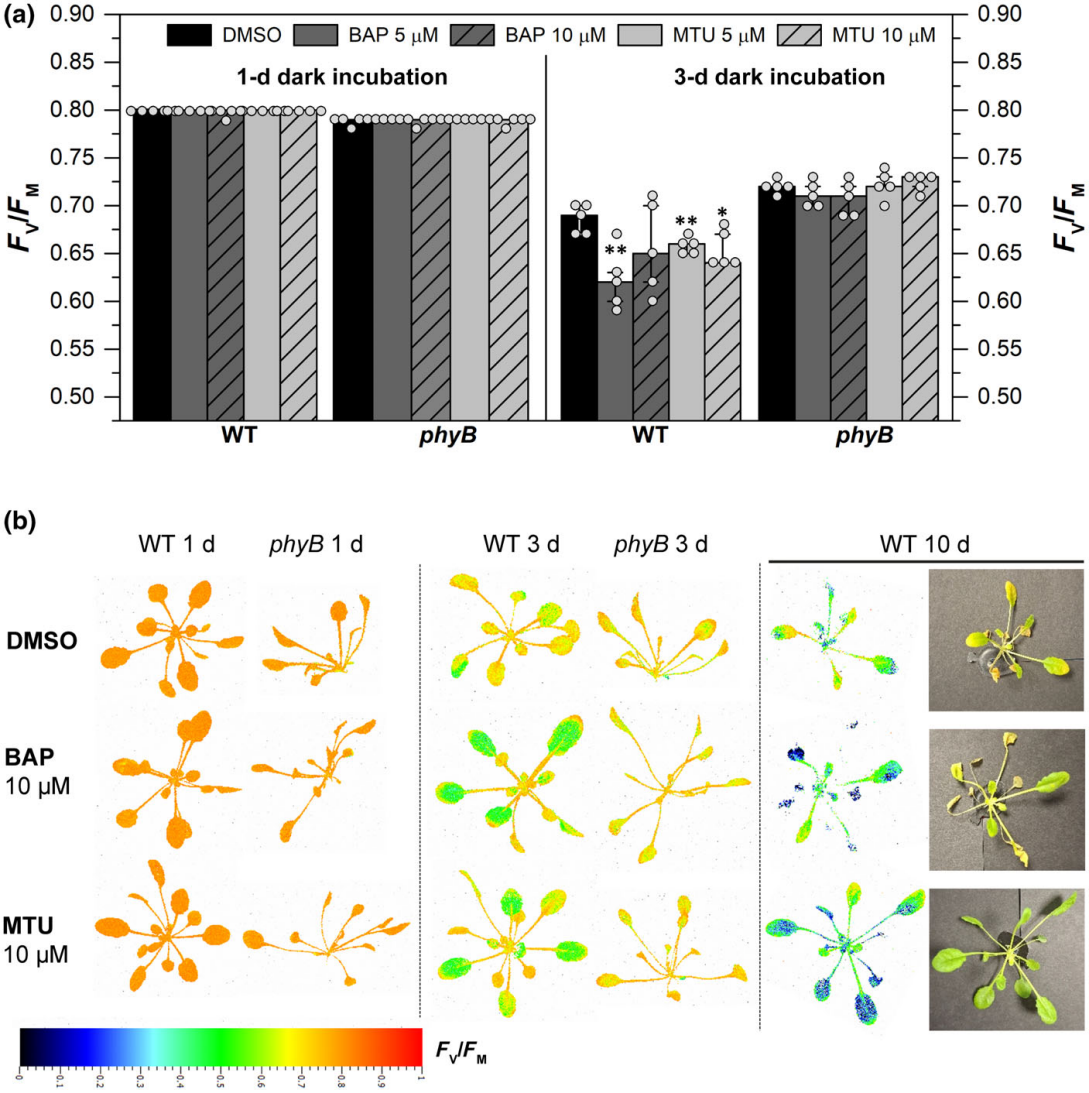
Effects of cytokinin (CK) treatments on *F*_V_/*F*_M_ in *Arabidopsis thaliana* intact plants during darkness. (a) Maximum quantum yield of Photosystem II (PSII) photochemistry (*F*_V_/*F*_M_) in hydroponically grown *Arabidopsis thaliana* Columbia‐0 (Col‐0) (wild‐type, WT) or *phyB* mutant (SALK_022035C) plants after 1 or 3 d in the dark. Plants were treated with mock (0.02% dimethylsulfoxide (DMSO)), benzylaminopurine (BAP), or 1‐(2‐methoxyethyl)‐3‐(1,2,3‐thiadiazol‐5‐yl)urea (MTU) at concentrations of 5 or 10 μM. Asterisks indicate a statistically significant difference (Student's *t*‐test; *P* < 0.05) between mock‐ and CK‐treated samples (BAP or MTU, *n* = 5; medians and quartiles are presented, together with individual data points) within the same genotype. (b) Imaging of *F*_V_/*F*_M_ in representative mock‐ (0.02% DMSO) and CK‐treated (10 μM BAP or MTU) plants after 1‐ and 3‐d dark incubations (WT and *phyB*) and after 10‐d incubation (WT only).

In order to determine whether the CK‐induced downregulation of PSII photochemistry and its dependence on phyB represent a general mechanism, we performed experiments with intact plants. In WT plants, CK treatment (particularly BAP) led to a significant decrease in *F*_V_/*F*_M_ after 3 d of dark incubation, whereas no effect was evidenced after 1 d (Fig. 8a,b). In the *phyB* mutant, however, *F*_V_/*F*_M_ remained close to control values under both CK treatments at both time points, indicating that the CK‐induced downregulation of PSII photochemistry requires phyB also in intact plants under the conditions of prolonged darkness.

Consistent with the detached‐leaf assays, CKs – especially MTU – attenuated dark‐induced senescence at later stages in intact WT plants (10 DAD), as reflected by better retention of photosynthetically active (green) leaves compared with DMSO‐treated controls (Fig. 8b). At the same time, *F*_V_/*F*_M_ values dropped significantly, confirming that the phyB‐dependent CK downregulation of PSII photochemistry during prolonged darkness is a more general phenomenon.

## Discussion

Our results reveal a multifaceted role of CKs in modulating photosynthetic processes and delaying leaf senescence under prolonged darkness. While the antisenescence and photosynthesis‐promoting effects of CKs are relatively well‐documented (Cortleven & Schmülling, 2015), their influence on PSII photochemistry in leaves during prolonged darkness or shading, that is before the onset of dark‐induced senescence, has been largely unexplored. Under normal conditions, CKs help maintain the structure and function of the photosynthetic apparatus, including preserving chloroplast integrity by protecting key components of the photosystems (Brenner *et al*., 2012; Cortleven *et al*., 2014; Skalák *et al*., 2019). Under stress conditions, CKs have also been reported to exert a strong positive effect on photosynthetic function (Kučerová *et al*., 2020; Nisler *et al*., 2023).

Here, we show that the impact of CKs on PSII photochemistry shows a dual‐phase response. At later stages of senescence (from 7 DAD), CKs effectively delayed the decline in PSII functionality, in agreement with our previous results. However, at earlier stages of senescence (2 and 5 DAD), we observed the suppression of PSII photochemistry in CK‐treated leaves (with similar effects for both BAP and MTU treatments; Figs 1b, 2a), despite the retention of Chl (Fig. 1a) and LHCs (Fig. 5c). A possible explanation is that CK treatment may transiently alter the organization and/or function of the photosynthetic complexes to ensure an effective and safe restart of photosynthesis upon re‐illumination. Due to the CK‐mediated preservation of photosynthetic components in the thylakoid membranes, the primary photochemistry can be activated immediately upon re‐illumination; however, it can concurrently increase the risk of photo‐oxidative damage, especially before the biochemical photosynthetic processes in the chloroplast stroma become fully re‐established after prolonged darkness. The observed reduction in *F*_V_/*F*_M_ during prolonged darkness reflects the activation of energy dissipation mechanisms (of a different type than in the mock‐treated leaves, Fig. 2b) that prevent possible overexcitation of PSII after re‐illumination, especially under high‐light conditions (Figs 2d, 7c). In this context, CKs can influence the distribution of excitation energy between PSII and PSI and stimulate CET around PSI (Fig. 2c), while simultaneously preserving the integrity of both photosystems, as demonstrated by several lines of evidence.

The stimulation of CET around PSI in CK‐treated leaves is particularly noteworthy. CET plays a crucial role in balancing ATP/NADPH production and suppressing chloroplastic ROS formation, especially under stress conditions (Joliot & Johnson, 2011). Both CKs upregulated genes of the NDH complex involved in CET (Fig. S6) and markedly increased CET in WT leaves (Figs 2c, 7b). MTU treatment was recently shown to activate CET also in intact wheat plants (especially under stress conditions; Nisler *et al*., 2023), suggesting that the CET stimulation might represent a more general effect of CKs. In the CK‐treated WT leaves with the enhanced CET, a lower ROS accumulation (Fig. 2d) and decreased level of lipid peroxidation (Fig. 7c) were observed, which confirms the CK‐mediated protective role of CET from photo‐oxidative damage.

Our transcriptomic and proteomic analyses showed that the effects of both CKs at the transcriptomic and proteomic levels partly overlapped, particularly in categories related to the translational machinery and the promotion of photosynthesis, light sensing, and photomorphogenesis. At the protein level, we observed clear positive effects on the photosynthetic apparatus and chloroplast architecture, complemented by enhanced chloroplast translation and ribosome biogenesis, as well as adaptive changes of metabolic processes that reflect adjustments to prolonged darkness (Figs 4, S5). Together with the transcriptomic changes (Fig. 3e,f), these findings are consistent with a role of CKs in preserving chloroplast functionality and delaying dark‐induced senescence.

Upregulation of Chl biosynthesis enzymes (CHLI1/2, CHLD, CHLH, and CRD1) and chloroplast assembly proteins (HCF101, TIC56, and PSA3) aligns with the transcriptional activation of photosystem‐related genes (and the downregulation of Chl catabolic process‐related genes; see Fig. 3d), that is processes linked to Chl metabolism and chloroplast organization. In addition, the induction of chloroplastic translation and ribosome biogenesis proteins highlights CK's role also in maintaining translational capacity during darkness. This observation complements the transcriptomic enrichment of ribosomal and translational GO categories and supports the idea that CK stabilizes plastid gene expression and ribosome assembly, thereby preserving chloroplast functionality. Altogether, these changes support the interpretation that CKs sustain chloroplast integrity, including photosynthesis apparatus, even during darkness, possibly preparing leaves for rapid reactivation of photosynthesis upon re‐illumination. This aligns with the concept of CK‐mediated suppression of ‘accidental senescence’ proposed by Ueda *et al*. (2020), where CKs prevent unnecessary degradation of cellular structures when light availability is uncertain.

Another interesting effect of CK observed on the proteomic level is the modulation of amino acid metabolic processes, especially in response to MTU treatment (Fig. 4c). The upregulation of enzymes linked to BCAA metabolism (amino acids Val, Leu and Ile; Table S8), together with the reduced abundance of glutamine synthetase GLN1;1, points to a coordinated metabolic reprogramming. Enhanced BCAA biosynthetic and transaminase activity may provide alternative substrates for respiration and redox balance under carbon‐limited conditions (Araújo *et al*., 2010; Binder, 2010), while suppression of glutamine synthetase could reflect a transient reduction in nitrogen assimilation and catabolism, consistent with delayed senescence (Li *et al*., 2006).

We propose that the physiological state of our detached, darkened, and CK‐treated leaves is analogous to that described in the studies of Keech *et al*. (2007) and Law *et al*. (2018) in darkened plants, where metabolism was adjusted to rely on alternative substrates for minimal ATP production while maintaining the photosynthetic apparatus, ensuring a rapid transition back to photosynthesis upon light exposure. In this metabolically suppressed state, the production of BCAAs was promoted, leading to enhanced synthesis of acetyl‐CoA for the tricarboxylic acid (TCA) cycle (Law *et al*., 2018). Notably, our proteomic data corroborate this model, showing a significant increase in processes related to BCAA biosynthesis and the acetyl‐CoA biosynthetic process (Tables S6, S7).

One of our key findings is that the described CK effects during prolonged darkness are strongly phyB‐dependent. Previous studies have shown that FR supplementation and phyB inactivation enhance auxin biosynthesis and signaling (Leivar & Quail, 2011; Hersch *et al*., 2014), and auxin can antagonize CK signaling (Kieber & Schaller, 2018). In addition, shade (low R: FR) conditions have been associated with the induction of CKX, suggesting that light quality can modulate CK homeostasis (Ahres *et al*., 2021; Lei *et al*., 2022). While these mechanisms suggest that phyB status may influence CK homeostasis, our data indicate that phyB is also required for the full transcriptional and physiological execution of the CK response. Importantly, CK treatments retain transcriptional effects in *phyB*, albeit with modified amplitude, suggesting partial loss of CK‐driven transcriptional response. Our results show that the CK‐induced suppression of PSII photochemistry was pronouncedly attenuated in WT exposed to FR light and disappeared in the *phyB* mutant (Fig. 7a). The CK‐induced stimulation of CET was suppressed in *phyB* (Fig. 7b). Furthermore, the upregulation of *ARR5* transcript levels in the CK‐treated leaves was markedly attenuated in *phyB* (Fig. 6). This matches a model in which elevated PIF activity in *phyB* directly represses transcription of *ARR5* and other A‐type *ARRs* (Gautrat *et al*., 2024). However, in *phyB* we also observed strong downregulation of *HY5* and *CCA1*, and concomitantly also *CAB1* transcript levels (Fig. 6). Importantly, these changes do not indicate a global impairment of CK signaling competence in *phyB*, but rather a selective disruption of phyB‐dependent transcriptional modules linked to light signaling and photomorphogenesis in general.

Based on previous evidence for ARR4–phyB interactions, we propose that CK signaling may stabilize phyB in its active Pfr form, potentially via ARR4 or related mechanisms (Sweere *et al*., 2001; Hanano *et al*., 2006; Mira‐Rodado *et al*., 2007). Yet, another possibility arises from the recent publication of Gautrat *et al*. (2024), who observed that phyB fine‐tunes the CK output via PIFs – when phyB is active (Pfr), it suppresses PIF activity, therefore alleviating the pressure on A‐type ARR‐driven negative loop (Gautrat *et al*., 2024).

Such direct or indirect CK‐induced stabilization of the Pfr form of phyB not only prevented triggering dark‐induced senescence but also induced metabolic reprogramming toward the ‘standby’ mode. This reprogramming is probably associated with an accumulation of reducing agents in the chloroplast stroma, resulting in the nonphotochemical reduction of the PQ pool and downregulation of PSII photochemistry. The increased nonphotochemical reduction of the PQ pool in darkness has been previously observed under conditions of reduced mitochondrial respiration (Havaux, 1996; Nellaepalli *et al*., 2012) and is thought to partially compensate for the loss of ATP production in the absence of active photosynthesis. Although a protective role of PSII downregulation in preventing PSI photoinhibition has been reported (Sonoike, 2011), this mechanism has, to our knowledge, not yet been discussed in the context of impeding senescence and phyB signaling. Taken together, our data are consistent with a scenario in which transient, phyB‐dependent downregulation of PSII via nonphotochemical reduction of the PQ pool preserves – or even enhances – the readiness of the photosynthetic apparatus for rapid reactivation upon re‐illumination, while minimizing oxidative stress.

**Fig. 9:**
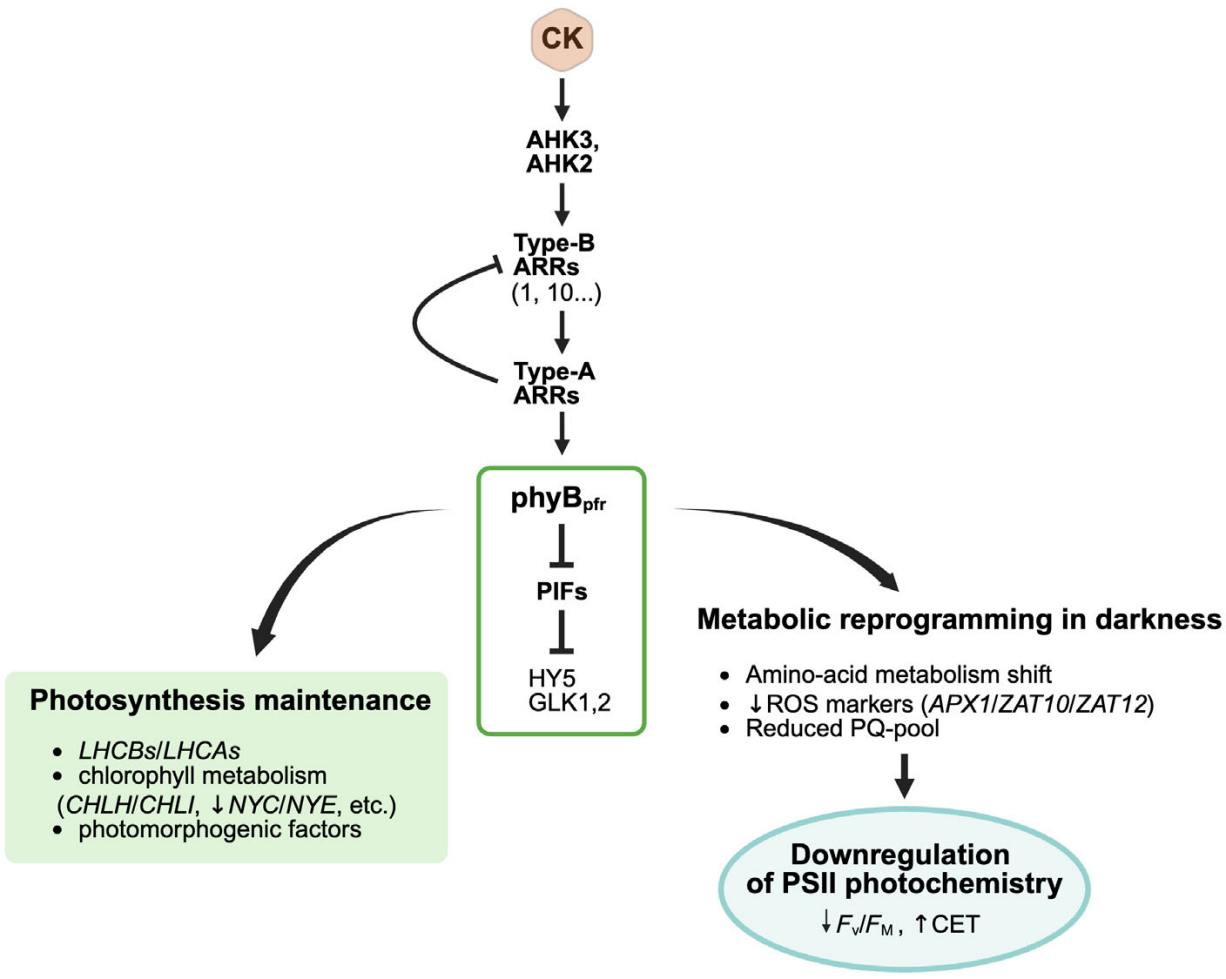
Cytokinin (CK)–phyB signaling modulates Photosystem II (PSII) photochemistry during prolonged darkness. We propose that, at the onset of darkness, CK perception via AHK2/AHK3 receptors activates a signaling module that transiently sustains phyB activity. By inhibiting the dark conversion of phyB from its active form (phyB_pfr_) to the inactive form (phyB_pr_), CKs partially mimic light and maintain a photomorphogenic state. Active phyB_pfr_ prevents the accumulation of PHYTOCHROME‐INTERACTING FACTORS (PIFs), relieving repression of HY5/GLKs targets, and inhibiting dark‐induced decomposition of the photosynthetic apparatus (both photosynthetic pigments and proteins). In parallel, CKs promote a metabolic rearrangement to lower energy demands and to use alternative energy sources. This metabolic shift is associated with the downregulation of reactive oxygen species markers and PQ‐pool reduction in darkness. The more reduced PQ‐pool downregulates PSII photochemistry, facilitating a safe and efficient transition from dark to light upon re‐illumination and also limiting photo‐oxidative damage under high light. Solid arrows indicate positive regulatory links, whereas blunt‐ended lines indicate negative regulation/repression. Curved arrows indicate downstream processes regulated by the CK‐phyB module. Created with BioRender (https://BioRender.com/ts8q17h).

Based on our findings, we propose a model which integrates CK‐ and phyB signaling in mature leaves under prolonged darkness (Fig. 9). In this model, the CK‐stabilization of active phyB suppresses PIFs, permitting HY5/GLK to sustain photosynthetic apparatus, and a parallel phyB‐dependent metabolic readjustment allows leaves to survive under limiting light. This way, CK‐phyB module acts as a photomorphogenic signal: it partially mimics light by stabilizing the photosynthetic machinery, promoting chloroplast translation, and transiently downregulating PSII primary photochemistry while buffering ROS production. This may, under natural conditions, help plants cope with day–night transitions or rapid fluctuations in light intensity (e.g. following prolonged shading). Beyond refining our understanding of CK–light crosstalk at the molecular level, our findings suggest that hormonal control of phytochrome activity could be exploited to fine‐tune stress resilience and stay‐green traits in crops.

## Competing interests

JN and MS are authors of several patents claiming protection of MTU. The remaining authors declare that the research was conducted in the absence of any commercial or financial relationships that could be seen as a potential conflict of interest.

## Author contributions

MŠ, OP and MS contributed to the conceptualization. VK, ZK, OP, IC and FZK contributed to the formal analysis. VK, ZK, PR, JS, MH, MR and TV contributed to the data acquisition and methodology. RL, JN, JH and MS contributed to the resources. OP and MŠ wrote the original draft. OP, MŠ, JH and MS supervised the study. ZK, JS, JN, VK, MR, TV and IC contributed to the review & editing. VK and ZK contributed equally to this work. All authors approved the manuscript.

## Disclaimer

The New Phytologist Foundation remains neutral with regard to jurisdictional claims in maps and in any institutional affiliations.

## Supporting information


**Fig. S1** Reversibility of *F*_V_/*F*_M_ decrease upon re‐illumination.
**Fig. S2** PCA analysis of RNA‐seq data.
**Fig. S3** GO enrichment network of pathways responding to BAP/MTU treatments.
**Fig. S4** Cytokinin root inhibition assay and signaling in response to BAP/MTU treatments.
**Fig. S5** Proteomic analysis of BAP‐treated leaves of *Arabidopsis thaliana*.
**Fig. S6** RT‐qPCR analysis in *Arabidopsis thaliana* wild‐type leaves.
**Fig. S7** Cytokinin‐dependent transcriptional changes in light‐signaling pathways.
**Fig. S8** Spectrum of far‐red light used in the study.
**Table S1** List of RT‐qPCR primers.

**Table S2** List of common genes that were responding under all experimental conditions.

**Table S3** GO enrichment analysis of genes responding to CK treatments (6, 48 h).

**Table S4** GO enrichment analysis of genes responding to CK treatments (30 min).

**Table S5** Differential protein analyses: All proteins responding to BAP and MTU treatments.

**Table S6** Classification of proteins responding to MTU treatment.

**Table S7** Classification of proteins responding to BAP treatment.

**Table S8** GO enrichment analysis of proteins responding to CK treatments.
Please note: Wiley is not responsible for the content or functionality of any Supporting Information supplied by the authors. Any queries (other than missing material) should be directed to the *New Phytologist* Central Office.

## Data Availability

RNA‐Seq data are openly available at National Center for Biotechnology Information (NCBI) Sequence Read Archive (SRA) (https://www.ncbi.nlm.nih.gov/sra) under the SRA accession PRJNA1210121. The mass spectrometry proteomics data have been deposited to the ProteomeXchange Consortium (http://proteomecentral.proteomexchange.org) via the PRIDE partner repository (Perez‐Riverol *et al*., 2022) with the dataset identifier PXD047787. The data supporting the findings of this study are provided in the Supporting Information of this article, specifically in Tables S2–, S8, which include transcriptomic/proteomics datasets and associated downstream analyses. Additional raw data not included in the supplementary files are available at Zenodo, doi: 10.5281/zenodo.17700840.
