# Supplementary material for: Cytokinin downregulates Photosystem II photochemistry during prolonged darkness in a phytochrome B‐dependent manner
Source: New Phytol. 2026 Apr 24;250(6):3846–66. doi: 10.1111/nph.71224 (PMC13193411; doi:10.1111/nph.71224)
Supplement: Supplementary file 1 — Fig. S1 Reversibility of F V/F M decrease upon re‐illumination. Fig. S2 PCA analysis of RNA‐seq data. Fig. S3 GO enrichment network of pathways responding to BAP/MTU treatments. Fig. S4 Cytokinin root inhibition assay and signaling in response to BAP/MTU treatments. Fig. S5 Proteomic analysis of BAP‐treated leaves of Arabidopsis thaliana. Fig. S6 RT‐qPCR analysis in Arabidopsis thaliana wild‐type leaves. Fig. S7 Cytokinin‐dependent transcriptional changes in light‐signaling pathways. Fig. S8 Spectrum of far‐red light used in the study. Table S1 List of RT‐qPCR primers. [file NPH-250-3846-s007.pdf]

New Phytologist

***New Phytologist* Supporting Information**

Article title: Cytokinin down-regulates photosystem II photochemistry during prolonged darkness in a phytochrome B-dependent manner

Authors: Veronika Kábrtová, Zuzana Kučerová, Ivo Chamrád, René Lenobel, Pavel Roudnický, Jan Skalák, Martin Hudeček, Filip Zavadil Kokáš, Marek Rác, Tereza Vánská, Jaroslav Nisler, Jan Hejátko, Miroslav Strnad, Martina Špundová, and Ondřej Plíhal

Article acceptance date: 31 March 2026

The following Supporting Information is provided in this file:

Fig. S1 Reversibility of F_V/F_M decrease upon re-illumination

Fig. S2 PCA analysis of RNA-seq data

Fig. S3 GO enrichment network of pathways responding to BAP/MTU treatments

Fig. S4 Cytokinin root inhibition assay and signaling in response to BAP/MTU treatments

Fig. S5 Proteomic analysis of BAP-treated leaves of *Arabidopsis thaliana*

Fig. S6 RT-qPCR analysis in *Arabidopsis thaliana* wild-type leaves

Fig. S7 Cytokinin-dependent transcriptional changes in light-signaling pathways

Fig. S8 Spectrum of far-red light used in the study

Table S1 List of RT-qPCR primers

Fig. S1 Reversibility of F_V/F_M decrease upon re-illumination. Maximum quantum yield of PSII photochemistry (F_V/F_M) in selected time points. Leaves from 33-day-old *Arabidopsis thaliana* wild-type plants (Col-0) were detached and incubated in the dark while submerged in solutions of 0.2% DMSO, 5 µM BAP, or 5 µM MTU for 2 days, and afterwards the F_V/F_M was measured (time point 1, indicated by the red arrow in the photoperiod chart above the bar graph). The leaves were subsequently re-illuminated by returning them to the standard growing conditions (8-h light (110 µmol photons m^{-2} s^{-1}; LED)/16-h dark cycle, and at 22/20 °C), and before the end of the photoperiod, F_V/F_M was measured again (time point 2). On the following day, approximately 3 h after the start of the photoperiod, the F_V/F_M was measured for the last time (time point 3). Different letters denote statistically significant differences between treatments for the given timepoint (Tukey's test, $P < 0.05$). Medians and quartiles are presented (n=5-9).

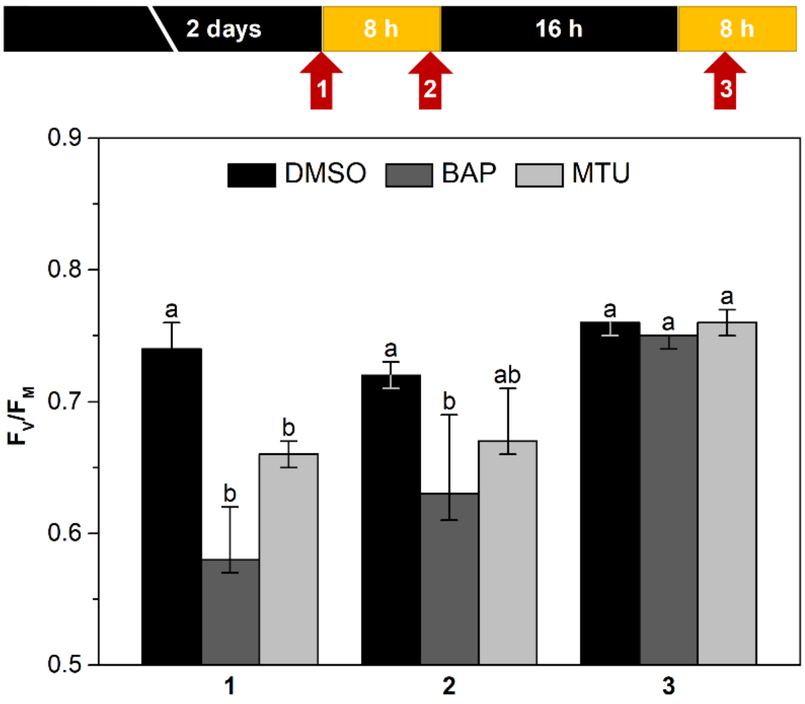

Fig. S2 PCA analysis of RNA-seq data. Principal component analysis (PCA) was performed using individual biological samples from a genome-wide transcriptomic analysis of detached, dark-incubated *Arabidopsis thaliana* Col-0 leaves treated with cytokinins or mock solution (DMSO control) for 6 h or 48 h. The cytokinin treatment and time points are indicated in the legend.

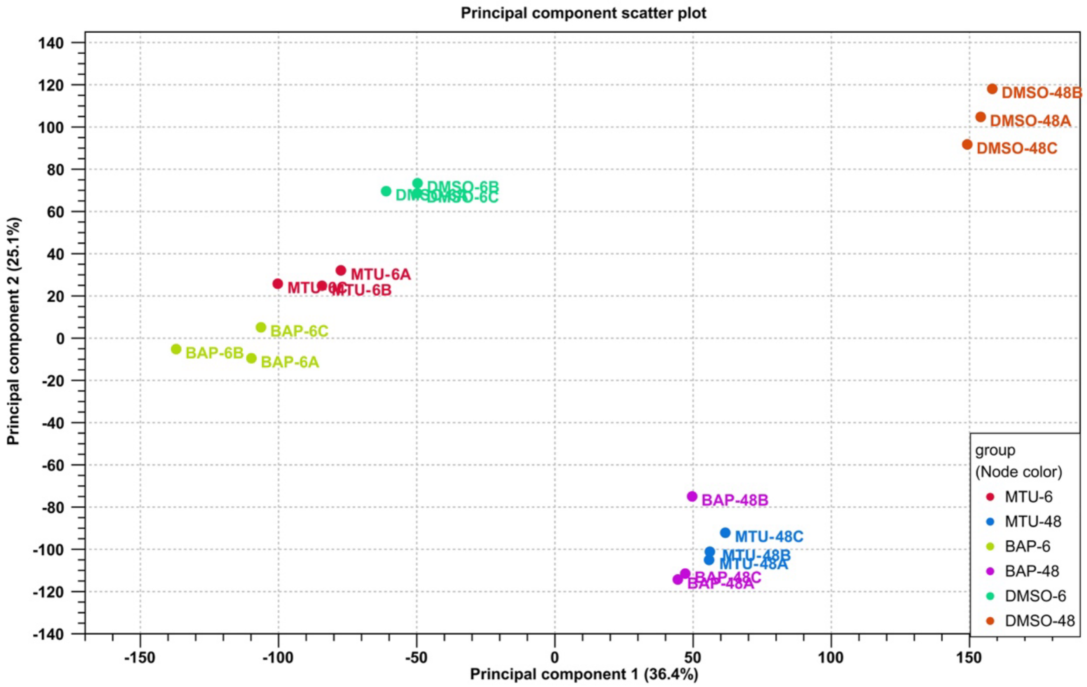

Fig. S3 Statistically enriched pathways responding to BAP (A) or MTU (B) treatments in detached, dark-incubated *Arabidopsis thaliana* leaves. AgriGo v2.0 database (Du *et al.*, 2010; Tian *et al.*, 2017) was used for Gene Ontology (GO) enrichment analysis from RNA-seq results. DEGs from *Arabidopsis thaliana* Col-0 leaves showing statistical differences in response to either short-term (6 h) or long-term (48 h) treatment with BAP or MTU were considered. Both upregulated and downregulated DEGs with a $|\log_2$ fold change$| > 1$ were included. Each box represents a significantly enriched GO biological process term; node color from yellow to red (Level1–Level9) indicates increasing statistical significance, whereas white nodes correspond to non-significant ancestor terms included to maintain the GO hierarchy. For each term, the GO identifier, term name, adjusted *P*-value (in brackets), and the number of genes in the input list and in the background (input/background) are shown. Arrows connect parent and child GO terms and indicate different types of GO relationships, as shown in the figure.

A

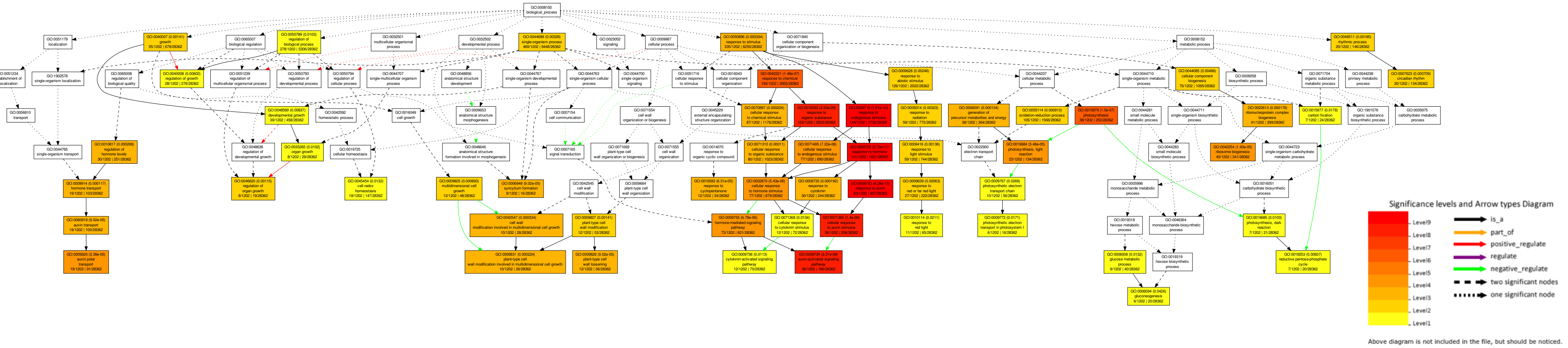

B

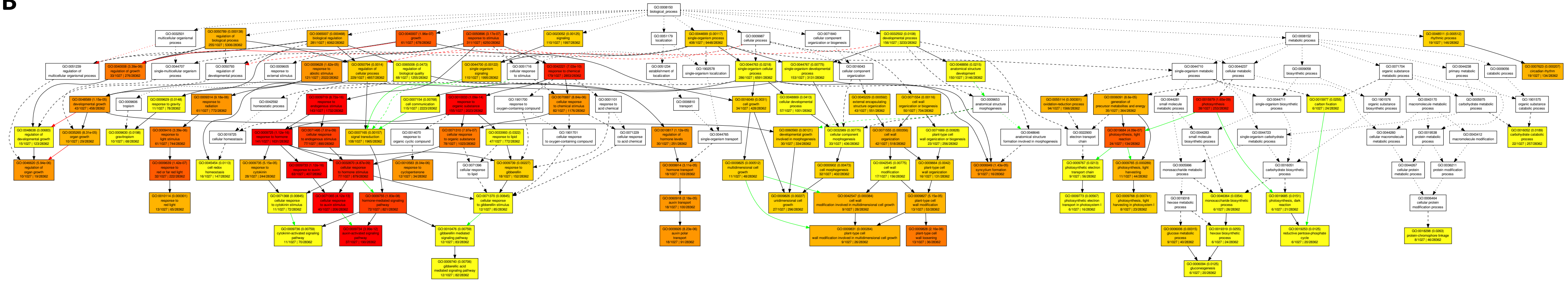

Fig. S4 Cytokinin root inhibition assay and signaling in response to BAP/MTU treatments. Phenotyping of *Arabidopsis thaliana* seedlings (**A**-**C**), *TCSv2:3XVENUS* reporter assay (**D**, **E**), and RT-qPCR analysis (**F**) after cytokinin treatments. (**A**, **B**, and **C**): The ½ MS plates were supplemented with different concentrations of MTU and BAP (0.1, 1, 5, or 10 µM in 0.2% DMSO) alongside a mock control group containing 0.2% DMSO. The plates were initially stored in a refrigerator for 3 days before being transferred to a phytotron environment. The phytotron was maintained at 120 µmol m^{-2} s^{-1}; 21°C with a 16/8h light/dark cycle. After a 10-day growth period in the phytotron, the root length (**A**) and count of lateral roots (**B**) were assessed in photographs (**C**, a representative image of a root inhibition assay) using ImageJ software. A minimum of 27 plants were evaluated for each experimental condition. In (**A**), BAP and MTU dose-response data were analyzed using two-way ANOVA followed by Tukey's multiple comparisons test ($P < 0.05$). Different letters indicate statistically significant differences among treatments. (**D**, **E**) Response of the *TCSv2:3XVENUS* reporter (Steiner *et al.*, 2020) to BAP and MTU treatments. Arabidopsis seedlings were treated for 15 hours with either BAP or MTU at a concentration of 10 µM. Mock-treated seedlings (0.2% DMSO) were used as controls. VENUS fluorescence was captured using a Zeiss LSM-900 confocal microscope. Scale bars = 20 µm. (**F**) Expression profiles of cytokinin signaling-related genes in *Arabidopsis thaliana* Col-0 seedlings following exogenous BAP or MTU treatments. Transcript levels were analyzed by qPCR at 1 hour, 3 hours, and 6 hours. BAP (dark gray bars) and MTU (light gray bars) were applied at a concentration of 1 µmol l^{-1}, and their effects were compared to mock-treated controls (black bars). In all charts, means ± SEM are presented, and Student's unpaired t-test was used (except A) to calculate the difference between mock and treatments ($^{*}P < 0.05$, $^{**}P < 0.01$, $^{***}P < 0.001$).

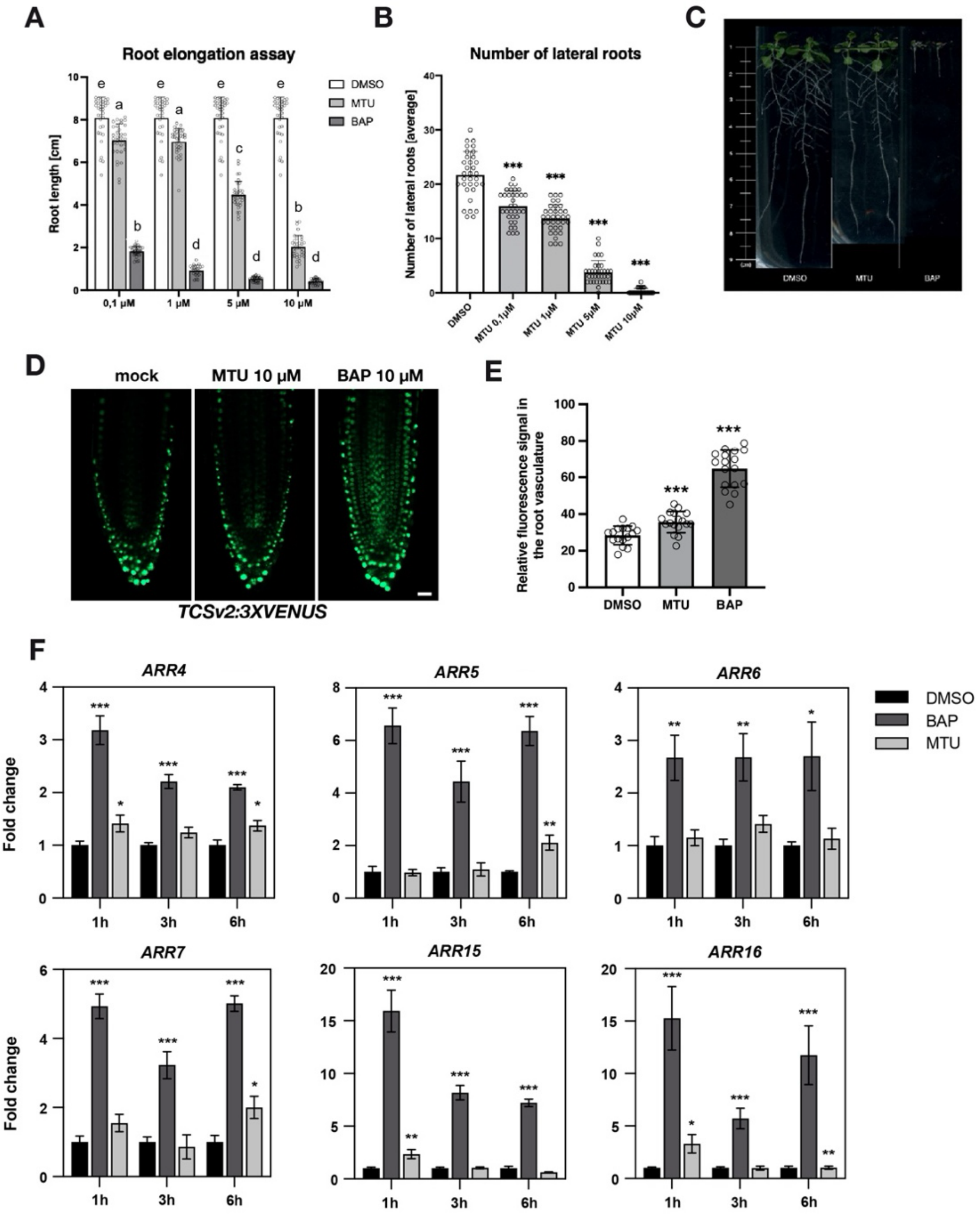
A
Root elongation assay
Root length [cm]
DMSO
MTU
BAP
0,1 µM
1 µM
5 µM
10 µM
B
Number of lateral roots
Number of lateral roots [average]
DMSO
MTU 0,1µM
MTU 1µM
MTU 5µM
MTU 10µM
C
DMSO
MTU
BAP
D
mock
MTU 10 µM
BAP 10 µM
TCSv2:3XVENUS
E
Relative fluorescence signal in the root vasculature
DMSO
MTU
BAP
F
ARR4
ARR5
ARR6
ARR7
ARR15
ARR16
Fold change
1h
3h
6h
DMSO
BAP
MTU

Fig. S5 Proteomic analysis of BAP-treated leaves of *Arabidopsis thaliana*. Detached *Arabidopsis thaliana* Col-0 leaves were incubated in mock solution (0.2% DMSO) or 5 µM BAP in darkness for 6 h (**A**) or 48 h (**B**). Volcano plots show proteins with significant differences in abundance after the indicated treatment. -Log10(Benjamini-Hochberg adjusted *P*-values) and Log_2(Fold change) are shown on the y- and x-axis, respectively. Non-axial lines denote cut-offs for statistically significant changes in adjusted *P*-value (<0.05) and fold change (>2). For visualization purposes, proteins with *P*-values of 0 were replaced with 1×10^{-5} to allow their display in the plot. Downregulated proteins are shown in blue and upregulated proteins in red. (**C**) Significantly enriched Gene Ontology (GO) terms connected with the differentially abundant proteins for BAP treatment. The x-axis displays the number of proteins related to the depicted GO term, the bubble size represents the term enrichment (relative overrepresentation of the given GO category compared to the reference proteome), and the bubble hue symbolizes the respective false discovery rate (FDR) value (applied cut-off < 0.05).

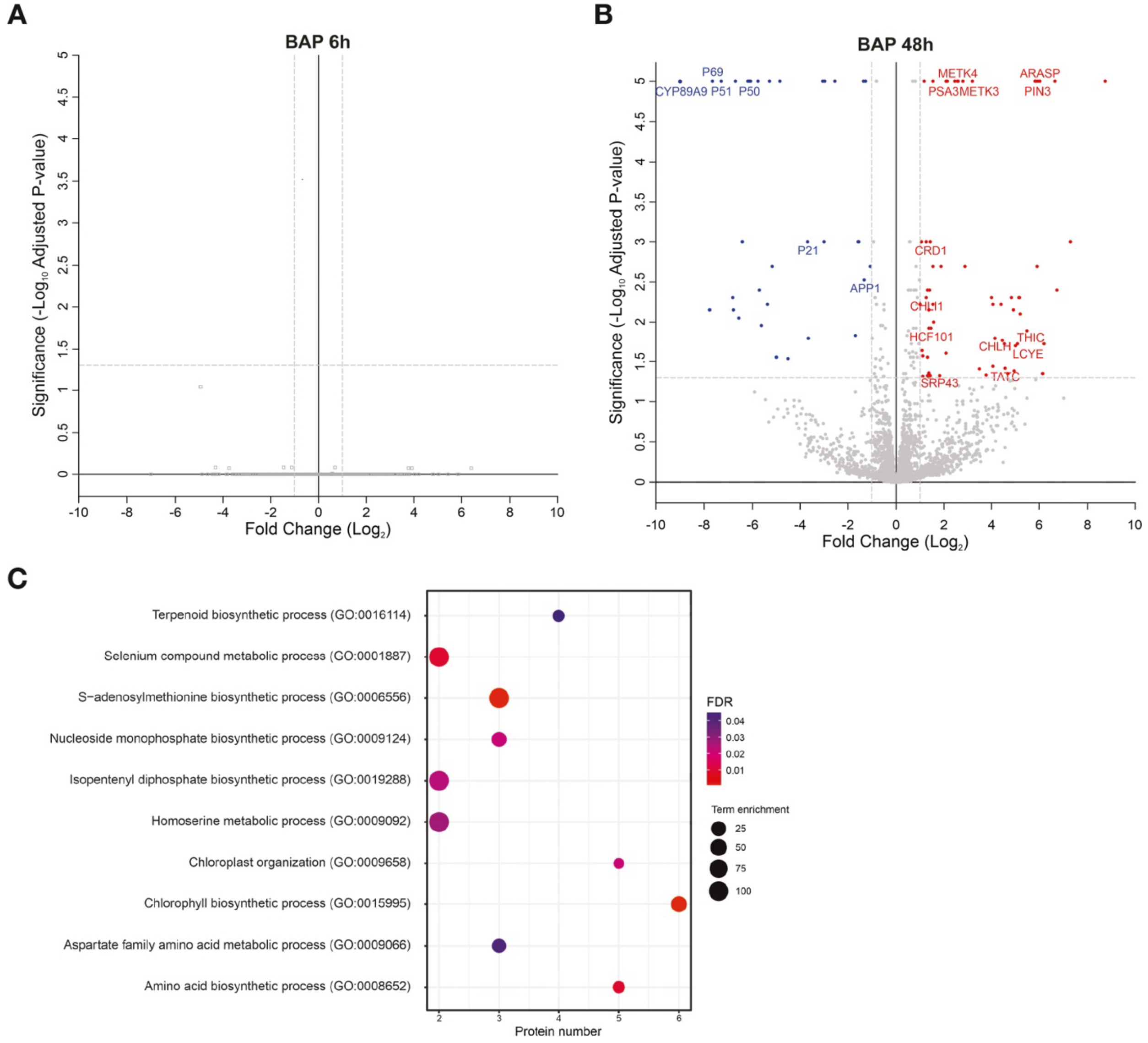

Fig. S6 Real-time qPCR analysis in *Arabidopsis thaliana* wild-type leaves. Transcript abundance of *PHYA, PHYB, NDHN*, *NDHM*, *CRR1*, and *WRKY6* was analyzed in leaves of 6-week-old *Arabidopsis thaliana* Col-0 plants treated with 5 µM BAP or 5 µM MTU, or mock solution (0.2% DMSO), after 2 days in darkness (2 DAD). In all charts, means ± SEM are presented, and Student's unpaired t-test was used to calculate statistical difference between mock- and cytokinin-treated samples ($^{*}P < 0.05$, $^{**}P < 0.01$, $^{***}P < 0.001$).

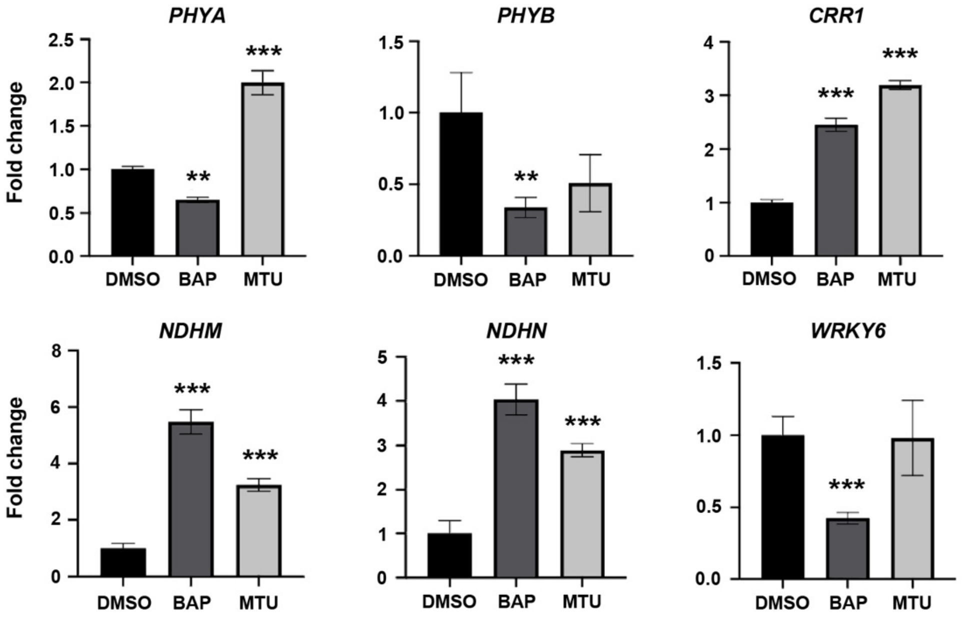

Fig. S7 Cytokinin-dependent transcriptional changes in light-signaling pathways. Heatmap of selected differentially expressed genes, including photoreceptors and their canonical downstream components, in darkened, detached leaves of *Arabidopsis thaliana* treated with 5 µM BAP or 5 µM MTU for 6 h or 48 h, compared with mock-treated controls (0.2% DMSO). Values are shown as log_2 expression changes relative to mock; the color bar indicates the scale.

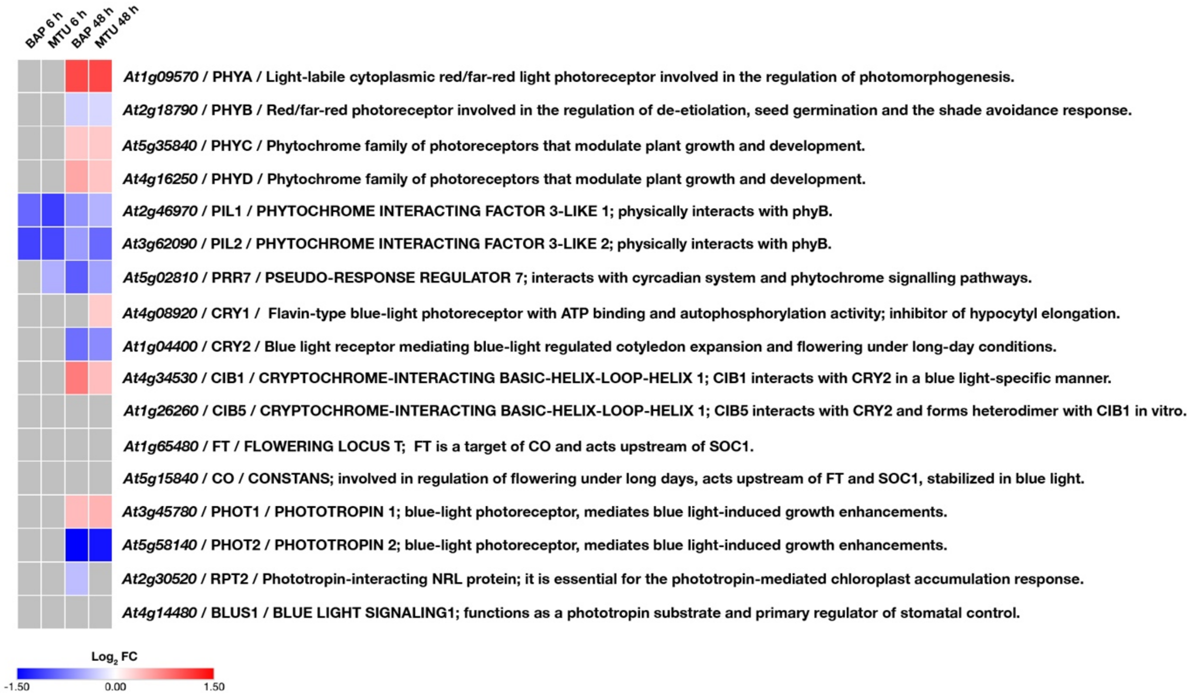

Fig. S8 Spectrum of far-red light used in the study. For all experiments with FR-light, the light was generated using the filter CG-RG-715-50.0M-2.5 (CVI Melles Griot, USA) in combination with white LED illumination.

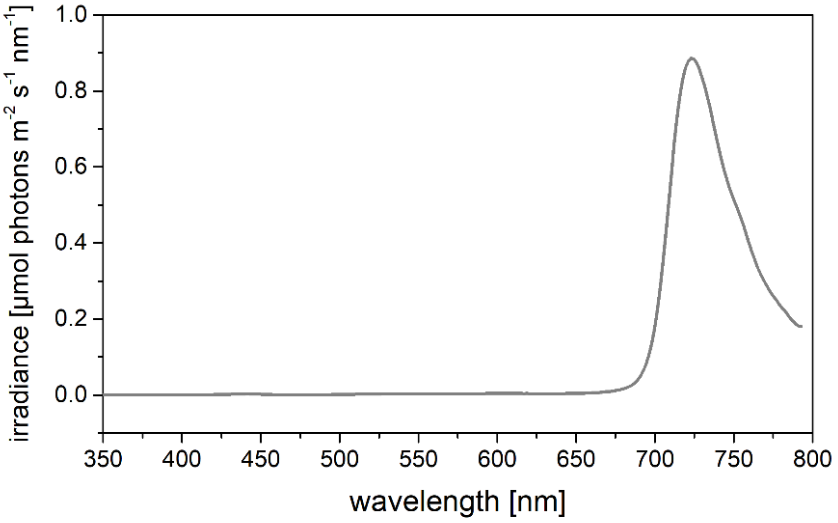

Table S1 List of RT-qPCR primers used in the study.

Gene	AGI No.	Primers 5´ - 3´
**Housekeeping genes (Endogenous controls)**		
Act7	*At5g09810*	CTAGAGACAGCCAAGAGCAGTTC<br>GTTTCATGGATTCCAGGAGCTTC
EF1α	*At5g60390*	TCCAGCTAAGGGTGCC<br>GGTGGGTACTCGGAGA
Target genes		
ARR4	*At1g10470*	GGTGGTATCGGAGGAATTG<br>ACGCCATCCACTATCTACCG
ARR5	*At3g48100*	TGCCTGGGATGACTGGATATG<br>CTCCTTCTTCAAGACATCTATCG
ARR6	*At5g62920*	TGCTCAGAGTATCTTCTTGCAA<br>AACCTTCAAATCCTCAAAACCG
ARR7	*At1g19050*	TACTCAATGCCAGGACTTTCAGG<br>TCTTTGAGACATTCTTGTATACGAGG
ARR15	*At1g74890*	CTGCTTGTAAAGTGACGACTGTTG<br>AGTTCATATCCTGTTAGTCCCGGC
ARR16	*At2g40670*	CGTAAACTCGTTGAGAGGTTGCTC<br>GCATTCTCTGCTGTTGTCACTTTG
NDHM	*At4g37925*	AGGATCGGACGTAGAACATTAC<br>GGTATTGAATTGTACACGAGGC
NDHN	*At5g58260*	TCCACCAGAAGTTGATTACAGG<br>TAGGACGAAGAGAGGGAAGTAA
WRKY6	*At1g62300*	CTCCGACAAGAAATCTAGG<br>TTTTTCGCACGCTTATCTTC
CRR1	*At5g52100*	ATCACGACATCTCAAACCTTCT<br>CGCTCTACCTATTTCCTTAGC
PHYA	*At1g09570*	CGTTTAATTGAGACTGCTACGG<br>GTGAGGAAATGCTTCCCGATT
PHYB	*At2g18790*	AAAAGATGTTGTGGAGTGGTTG<br>TTGATTTCTTTCGCAGTGTGAG
CCA1	*At2g46830*	TCGAAAGACGGGAAGTGGAACG<br>GTCGATCTTCATTGGCCATCTCAG
APX1	*At1g07890*	GATGTCTTTGCTAAGCAGATGG<br>GAGTTGTCGAAGATTAGAGGGT
LHCB1.3 (CAB1)	*At1g29930*	TTTGGTTCAAGGCCGGTTCAC<br>CCGGGGTAAAGCAAGTCCTC
HY5	*At5g11260*	AAACAGAGTGAAAGACTTGGAG<br>CGTACATGTCTAAGCATCTGG

Supplementary References

Du Z, Zhou X, Ling Y, Zhang Z, Su Z. 2010. agriGO: a GO analysis toolkit for the agricultural community. *Nucleic Acids Res* **38**(Web Server issue): W64-70.

Steiner E, Israeli A, Gupta R, Shwartz I, Nir I, Leibman-Markus M, Tal L, Farber M, Amsalem Z, Ori N, et al. 2020. Characterization of the cytokinin sensor TCSv2 in Arabidopsis and tomato. *Plant Methods* **16**(1): 152.

Tian T, Liu Y, Yan H, You Q, Yi X, Du Z, Xu W, Su Z. 2017. agriGO v2.0: a GO analysis toolkit for the agricultural community, 2017 update. *Nucleic Acids Res* **45**(W1): W122-W129.

List of Abbreviations (used in the main text)

- **AHK** – Arabidopsis histidine kinase (cytokinin receptor)
- **AHP** – Arabidopsis histidine-containing phosphotransfer protein
- **ARR** – Arabidopsis response regulator
- **BAP** – 6-benzylaminopurine
- **BCAA** – branched-chain amino acid(s)
- ***cZ*** – *cis*-zeatin
- **CET** – cyclic electron transport (around PSI)
- **Chl** – chlorophyll
- **CK(s)** – cytokinin(s)
- **CKX** – cytokinin oxidase/dehydrogenase
- **CRE1/AHK4** – CYTOKININ RESPONSE 1 / ARABIDOPSIS HISTIDINE KINASE 4
- **DAD** – days after leaf detachment and darkening
- **DEG** – differentially expressed gene
- **DMSO** – dimethyl sulfoxide
- **ETRI** – electron transport rate through PSI
- **ETRII** – electron transport rate through PSII
- F_o – minimal chlorophyll fluorescence in the dark-adapted state
- F_m – maximal chlorophyll fluorescence in the dark-adapted state
- F_m' – maximal chlorophyll fluorescence in the light-adapted state
- F_v/F_m – maximum quantum yield of PSII photochemistry
- F^t – steady-state chlorophyll fluorescence at time *t*
- **FR** – far-red (light)
- **GO** – Gene Ontology
- **HY5** – ELONGATED HYPOCOTYL 5

- **iP** – isopentenyladenine
- **IPT** – isopentenyl transferase
- **KEGG** – Kyoto Encyclopedia of Genes and Genomes
- **LHC** – light-harvesting complex
- **LHCA** – light-harvesting chlorophyll a/b-binding protein of PSI
- **LHCB** – light-harvesting chlorophyll a/b-binding protein of PSII
- **LOG** – LONELY GUY phosphoribohydrolase
- **MDA** – malondialdehyde
- **MTU** – 1-(2-methoxyethyl)-3-(1,2,3-thiadiazol-5-yl)urea
- **NAD(P)H** – nicotinamide adenine dinucleotide (phosphate), reduced form
- **NDH** – chloroplast NAD(P)H dehydrogenase complex
- **OE** – overexpressor / overexpression line
- **PAR** – photosynthetically active radiation
- **PEP** – plastid-encoded RNA polymerase *[pokud je v textu]*
- **phyA, phyB** – phytochrome A, phytochrome B
- **PIF** – PHYTOCHROME-INTERACTING FACTOR
- **POR** – protochlorophyllide oxidoreductase
- **PQ** – plastoquinone
- **PSI** – photosystem I
- **PSII** – photosystem II
- **SAM** – S-adenosyl-L-methionine
- ***tZ*** – *trans*-zeatin
- **WT** – wild type
- Φ_P – effective quantum yield of PSII photochemistry
- Φ_{NPQ} – quantum yield of regulatory non-photochemical quenching
- $\Phi_{f,D}$ – quantum yield of constitutive non-regulatory energy dissipation
